# NMDA Receptors Enhance the Fidelity of Synaptic Integration

**DOI:** 10.1523/ENEURO.0396-20.2020

**Published:** 2021-03-02

**Authors:** Chenguang Li, Allan T. Gulledge

**Affiliations:** Department of Molecular and Systems Biology, Geisel School of Medicine at Dartmouth College, Hanover, NH 03755

**Keywords:** AMPA receptor, dendrite, EPSP-spike coupling, NMDA receptor, synapse, synaptic integration

## Abstract

Excitatory synaptic transmission in many neurons is mediated by two coexpressed ionotropic glutamate receptor subtypes, AMPA and NMDA receptors, that differ in kinetics, ion selectivity, and voltage-sensitivity. AMPA receptors have fast kinetics and are voltage-insensitive, while NMDA receptors have slower kinetics and increased conductance at depolarized membrane potentials. Here, we report that the voltage dependency and kinetics of NMDA receptors act synergistically to stabilize synaptic integration of EPSPs across spatial and voltage domains. Simulations of synaptic integration in simplified and morphologically realistic dendritic trees revealed that the combined presence of AMPA and NMDA conductances reduce the variability of somatic responses to spatiotemporal patterns of excitatory synaptic input presented at different initial membrane potentials and/or in different dendritic domains. This moderating effect of the NMDA conductance on synaptic integration was robust across a wide range of AMPA-to-NMDA ratios, and results from synergistic interaction of NMDA kinetics (which reduces variability across membrane potential) and voltage dependence (which favors stabilization across dendritic location). When combined with AMPA conductance, the NMDA conductance compensates for voltage-dependent and impedance-dependent changes in synaptic driving force, and distance-dependent attenuation of synaptic potentials arriving at the axon, to increase the fidelity of synaptic integration and EPSP-spike coupling across both neuron state (i.e., initial membrane potential) and dendritic location of synaptic input. Thus, synaptic NMDA receptors convey advantages for synaptic integration that are independent of, but fully compatible with, their importance for coincidence detection and synaptic plasticity.

## Significance Statement

Glutamate is an excitatory neurotransmitter that, at many synapses, gates two coexpressed receptor subtypes (AMPA and NMDA receptors). Computational simulations reveal that the combined synaptic presence of AMPA and NMDA receptors reduces variability in synaptic integration in response to identical patterns of synaptic input delivered to different dendritic locations and/or at different initial membrane potentials. This results from synergistic interaction of the slower kinetics and voltage dependence of NMDA receptors, which combine to enhance synaptic currents when synaptic driving forces are otherwise reduced (e.g., at depolarized membrane potentials or in distal, high-impedance dendrites). By stabilizing synaptic integration across dendritic location and initial membrane potential, NMDA receptors provide advantages independent of, but fully compatible with, their well-known contribution to synaptic plasticity.

## Introduction

In the vertebrate central nervous system, fast excitatory synaptic transmission is mediated primarily by the amino acid glutamate, which at many synapses gates two coexpressed ionotropic receptors: AMPA and NMDA receptors. While both receptor subtypes are gated by glutamate, and are permeable to monovalent cations, NMDA receptors are distinct in having slower kinetics, permeability for calcium, and voltage dependence because of channel blockade by extracellular magnesium ions at hyperpolarized membrane potentials (for review, see Iacobucci and Popescu, 2017). These features of NMDA receptors, which are highly conserved across phyla (Greer et al., 2017), are critical for their well-established role in gating associative synaptic plasticity, including certain types of long-term potentiation and depression (for review, see Lüscher and Malenka, 2012). NMDA receptors also influence synaptic integration, as the voltage dependence of NMDA receptors can promote linear (Cash and Yuste, 1998) or supralinear (Schiller et al., 2000) summation of EPSPs that occur in sufficient spatial and temporal proximity to relieve magnesium block of NMDA channels, and can reduce the voltage-dependent variability of mixed AMPA/NMDA synaptic currents (Diamond and Copenhagen, 1993; Connelly et al., 2016).

Because the input impedance of dendrites increases with distance from the soma, distal synapses generate larger, more depolarizing local dendritic EPSPs than do proximal synapses (Jaffe and Carnevale, 1999; Gulledge et al., 2005; Lajeunesse et al., 2013). Yet the ability of an individual AMPA-mediated EPSP to recruit synaptic NMDA conductance is tempered by its rapid kinetics (∼0.2- and 2.0-ms activation and decay, respectively), which, when combined with the limited local capacitance of narrow dendrites and dendritic spines, generates EPSPs that decay too rapidly to efficiently recruit slower activating (>2 ms) NMDA conductances (Stern et al., 1992; Gulledge et al., 2012). Instead, network-driven patterns of synaptic input interact within the dendritic tree based on their spatiotemporal relationships to recruit NMDA conductances (Polsky et al., 2009; Grienberger et al., 2014; Palmer et al., 2014; Cichon and Gan, 2015), especially when they occur in high-impedance dendritic branches or spines (Branco and Häusser, 2011; Gulledge et al., 2012; Harnett et al., 2012). NMDA conductances are also recruited when barrages involve repetitive activation of the same synapses, as the extended occupancy of glutamate sites on NMDA receptors (∼100 ms) “prime” them for immediate gating during subsequent AMPA-mediated local depolarization (Schiller et al., 2000; Polsky et al., 2004). With sufficient levels of synaptic activation, inward NMDA currents become self-sustaining “NMDA spikes” that amplify and prolong synaptic depolarization to generate supralinear summative events at the soma and axon (for review, see Antic et al., 2010; Branco and Häusser, 2010; Major et al., 2013; Grienberger et al., 2015).

While most prior studies examining the impact of synaptic conductance on the location and/or voltage dependence of synaptic transmission have focused on single synaptic events (Jaffe and Carnevale, 1999; Lajeunesse et al., 2013), we reasoned that the voltage dependency of NMDA receptors, combined with the distance-dependent electrotonic structure of dendritic trees, should reduce the variability of somatic drive (as measured as somatic depolarization and/or action potential generation) in response to spatiotemporal patterns of synaptic input occurring at different dendritic locations and/or from different initial membrane potential states. Here, we test this hypothesis using computational simulations to compare the impact of synaptic conductance (AMPA-only, NMDA-only, or both conductances together) on the integration of patterns of afferent input delivered to different dendritic locations and/or at different initial membrane potentials. Our results reveal that AMPA and NMDA conductances interact in a complementary fashion to stabilize synaptic integration and EPSP-spike coupling in ways that are independent of, yet fully compatible with, their well-established roles in coincidence detection and synaptic plasticity.

## Materials and Methods

### Code accessibility

The code used to generate the data in this paper is freely available online at http://modeldb.yale.edu/266802. The code is also available as Extended Data 1.

10.1523/ENEURO.0396-20.2020.ed1 Extended Data 1 Code used for key simulations in this article, also available online at http://modeldb.yale.edu/266802. Download Extended Data 1, ZIP file.

### Computational models

Simulations were made using NEURON 7.7 software (Carnevale and Hines, 2006; RRID: SCR_005393) run on MacOS 10.14 and/or on the Neuroscience Gateway portal (Sivagnanam et al., 2013). Morphologies used include “ball-and-stick” model neurons, consisting of somata connected to spinous dendrites of variable length, a hippocampal CA3 pyramidal neuron (Hemond et al., 2009), and a hippocampal dentate granule neuron (Schmidt-Hieber et al., 2007). Table 1 lists the dimensions and membrane parameters for all neuron morphologies. Active mechanisms consisted primarily of fast-inactivating voltage-gated sodium and delayed-rectifier potassium conductances (source codes available in ModelDB, entry 144385). Spines with neck resistances of 500 MΩ (Harnett et al., 2012) were positioned at 1-μm intervals along dendrites. Axons in all models were 2000 μm long, had diameters of 0.5 μm, and were attached to the soma via a 40-μm axon initial segment (AIS) that tapered from 2.0 μm (at the soma) to 0.5 μm (at the axon). Except for the AIS, voltage-gated conductances were evenly distributed in axons (i.e., axons were modeled as unmyelinated; see Table 1 for sodium and potassium channel densities in each neuronal compartment). Unless otherwise noted, ball-and-stick neurons included somata (20 × 10 μm) attached to a single tapering (5 μm to 1 μm) dendrite of variable length (0.2–1 mm; input resistances [R_N_] ranging from ∼125 to ∼310 MΩ). Because R_N_ can influence the impact of dendritic location on synaptic integration, for some simulations we delivered synaptic input to a 200-μm dendrite attached to a larger neuron typically having three additional 600-μm dendrites that did not receive synaptic input. In all simulations, models were initiated following a 1-s passive run to allow active conductances to reach a steady state. Simulations were run with time steps of 10 or 25 μs at a nominal temperature of 37°C.

**Table 1:** Model parameters

Neuron morphology	Compartment	Dimensions (l × w)	Number of segments	Active properties (max. conductance)
Ball-and-stick*	Soma	20 × 10 μm	3	Na^+^: 100 pS/μm^2^ K^+^: 100 pS/μm^2^
	Dendrite	200–1000 μm, tapering from 5 to 1 μm	1 per μm	Typically passive. Figure 3*C*: Na^+^ and K^+^ with linear decrease (100–10 pS/μm^2^).
CA3* and dentate granule neurons	Soma	As reconstructed	3	Na^+^: 100 pS/μm^2^ K^+^: 100 pS/μm^2^
	Dendrites	As reconstructed	1 per μm	Passive
All morphologies	Spines	Neck: 1 × ∼0.05 μm Head: 0.5 × 0.5 μm	1 1	As in parent dendritic compartment
	AIS	40 μm, tapering from 2 to 0.5 μm	40	Na^+^: 100 pS/μm^2^ (1st 5 μm) or 8000 pS/μm^2^ K^+^: 100pS/μm^2^ (1st 5 μm) or 2000 pS/μm^2^
	Axon	2000 × 0.5 μm	201	Na^+^: 300 pS/μm^2^ K^+^: 60 pS/μm^2^

**Figure 1.**
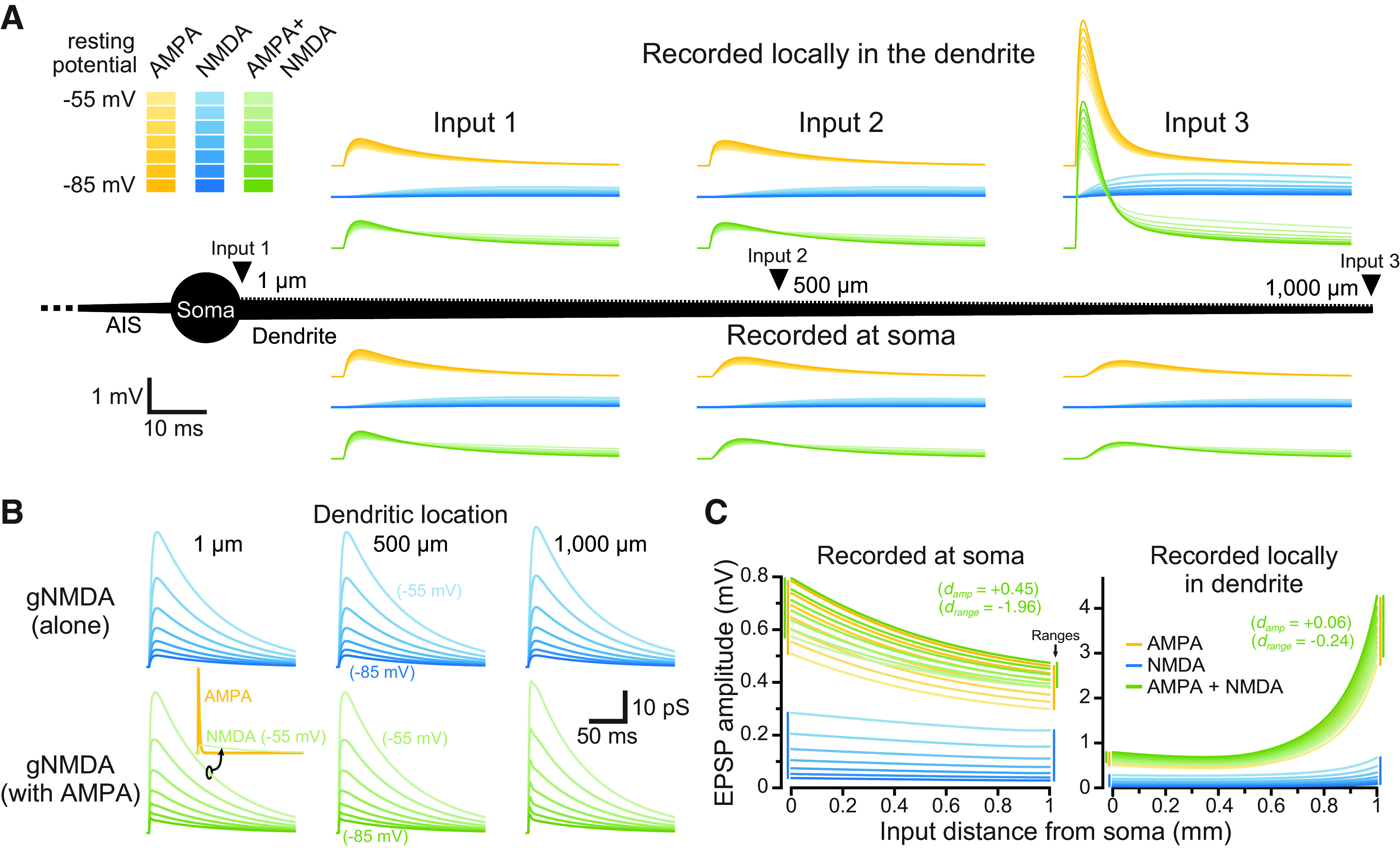
Impact of membrane potential and dendritic location on synaptic conductances and their resulting EPSPs. ***A***, Individual synaptic inputs were activated along the dendrite (1000 μm in length) of a passive ball-and-stick model neuron (black; not to scale). Shown are somatic (bottom) and local dendritic (top) EPSPs generated by synapses placed at three dendritic locations (1, 500, and 1000 μm from the soma), at seven different RMPs (−55 to −85 mV, as indicated by color depth). AMPA-only EPSPs are shown in yellow, NMDA-only synapses are shown in blue, and inputs having both AMPA and NMDA conductance are shown in green. ***B***, The NMDA conductances underlying the EPSPs shown in ***A***. Inset shows the 500 pS AMPA (yellow) and NMDA (green) conductances for the AMPA+NMDA input at −55 mV at the most proximal dendritic location (shown to scale). ***C***, Plots of the amplitudes of EPSPs having the indicated synaptic conductances measured at the soma (left) or locally at the site of synaptic input (right), versus dendritic location of the synapse. Synaptic conductances are color coded as in ***A*** for seven RMPs from −85 to −55 mV, at 5-mV intervals. Inset shows the effect sizes (*d*) for AMPA+NMDA EPSP amplitudes (across all dendritic locations and RMPs) and ranges (across all RMPs) relative to AMPA-only EPSPs. Colored vertical bars at margins indicate the ranges of amplitudes across all RMPs for the most proximal and most distal inputs.

Listed are the parameters for model neurons used in this study. General model parameters: R_M_ = 15 kΩ•cm^2^; C_M_ = 1 μF/cm^2^; R_i_ = 100 Ω•cm; E_pas_ = −55 to −85 mV, as indicated in text; time steps, 10 or 25 μs; nominal temperature 37°C. *The ball-and-stick model in Figure 1 and the CA3 neuron in Figure 8 are purely passive (including in somata and axons).

### Simulated synaptic inputs

In most simulations, synaptic conductances were located on spine heads. AMPA conductances (ModelDB entry 120798) typically had exponential rise and decay time constants of 0.2 and 2 ms, respectively, a reversal potential of 0 mV, and a maximum conductance of 500 pS, unless otherwise noted. NMDA inputs were based on the AMPA conductance, with added voltage dependence (as in Model DB entry 184725), and unless otherwise noted had nominal maximum conductances of 1 nS (typically reaching ∼46 pS during individual AMPA+NMDA synaptic events occurring from a membrane potential of −55 mV; see also Figs. 1*B*, 4*A*), reversal potentials of +5 mV, and exponential rise and decay time constants of 3 and 90 ms, respectively. Single synaptic potentials were monitored at the soma and at the dendritic site of synaptic input. For barrages of synaptic input, voltage responses were measured at the soma and at the mid-point of the dendritic span receiving synaptic input. During spike threshold tests, synaptic thresholds were determined by monitoring action potentials in the midpoint of the axon. During non-spike threshold tests, synaptic thresholds were determined as somatic events reaching 5 mV (for the dentate granule cell) or 3 mV (for the CA3 neuron) above the resting membrane potential (RMP).

Synaptic barrages of *n* synaptic activations, delivered to discrete dendritic compartments, were defined by sampling two random variables *n* times for a set of n pairs. The first random variable in the pair determined the site of the *n*th synaptic input and was chosen from a uniform distribution spanning integers 0–49 (for a 50-μm range), which was then applied to the dendritic segment of interest (i.e., from 620 to 669 μm from the soma). The second random variable, which determined synaptic timing, was chosen from a Gaussian distribution (width of 50 ms). Each pattern maintained a single ordered set of sampled pairs. The size of the set varied depending on the number n, but the content and order of the pairs did not change for a given pattern. For instance, within one pattern, barrages with n and *n + 1* synaptic activations were identical except for the *n + 1th* additional activation. These spatiotemporal patterns of synaptic input were iteratively moved along dendrites at 10- or 20-μm intervals to compare the effect of input location on local and somatic synaptic responses. Ten different randomized spatiotemporal patterns of synaptic input were used to determine the stability of EPSP-spike coupling over input pattern, dendritic location, and RMP. Nominal RMPs were set by uniformly adjusting the reversal potential for the passive leak conductance in all model compartments.

Variation of EPSP-spike coupling and somatic drive was quantified as coefficient of variation (CV; SD normalized by mean), which provides a relative measure of variability in synaptic thresholds across all dendritic locations and/or membrane potentials independent of response magnitudes (as opposed to measures of absolute range or SD, which are difficult to compare across models having different mean thresholds, and which may overestimate effective variability if functions [e.g., vs distance or voltage] are nonlinear). In the longest dendrites tested (the 1000-μm ball-and-stick and CA3 neurons), inputs to distal dendritic locations became so remote that threshold numbers of synaptic inputs rose exponentially to infinite values (i.e., beyond our maximum tested value of 10,000), as at very distal distances even voltage-clamping a 50-μm dendritic segment to 0 mV would fail to generate action potentials in the axon. Trials with AMPA-only inputs always hit this limit earliest (i.e., at slightly more proximal dendritic locations than other conductances), and so CVs for all conductances (AMPA-only, NMDA-only, or both AMPA and NMDA) were calculated only across dendritic locations where AMPA synaptic thresholds were measurable.

### Statistical analyses

**Figure 2.**
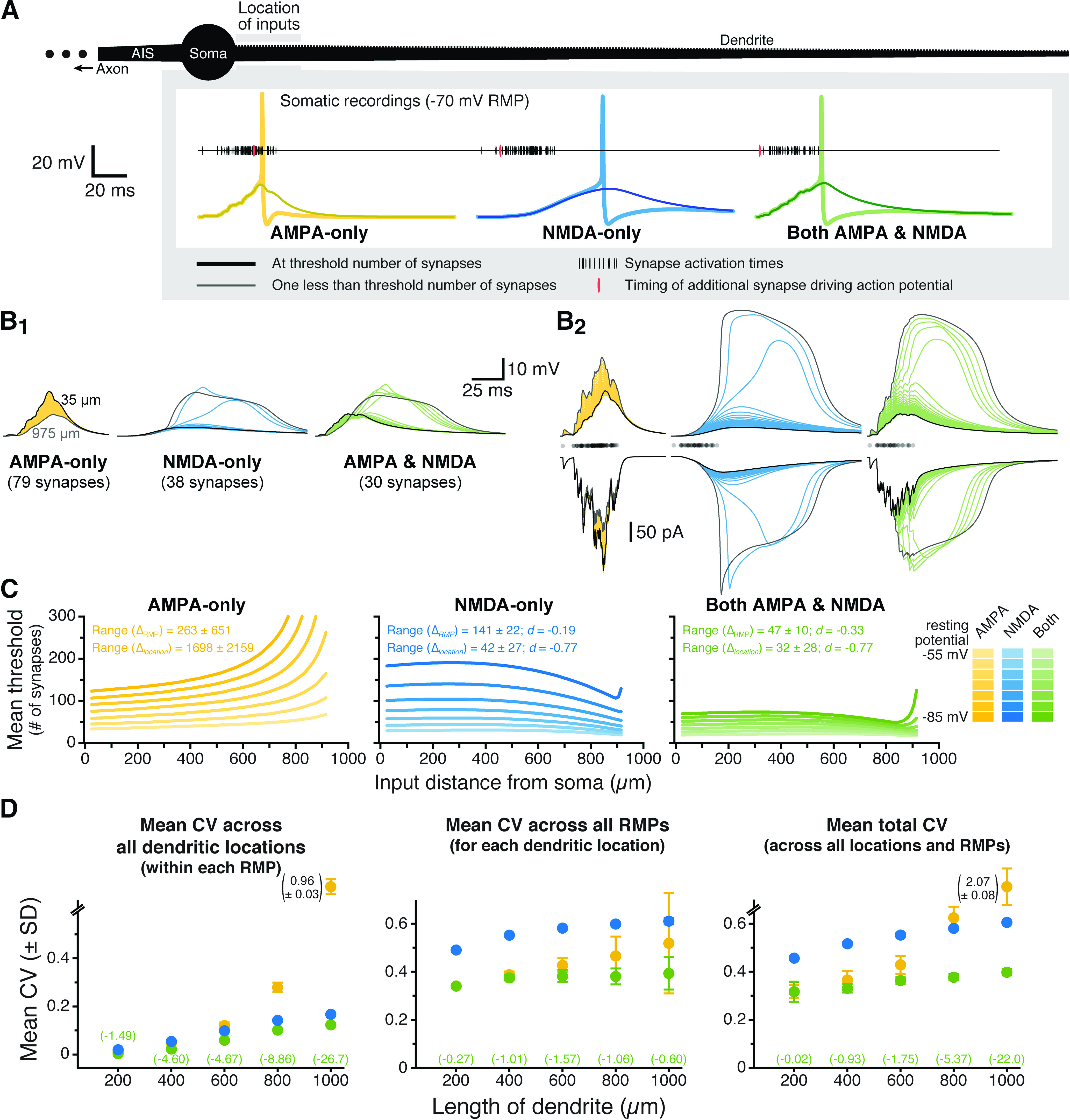
Combining synaptic AMPA and NMDA conductances stabilizes EPSP-spike coupling. ***A***, A spiny ball-and-stick neuron (black; 1000-μm dendrite) receiving progressively longer iterative trains of a spatiotemporal pattern of synaptic input. Just-threshold (thick traces) and just-subthreshold (thin traces) voltage responses in the soma for AMPA-only (yellow), NMDA-only (blue), or both AMPA and NMDA (green) synaptic conductances in response to inputs arriving within the first 50 μm of the dendrite. RMP is −70 mV. ***B***, Somatic (***B_1_***) and dendritic (***B_2_***, top) voltage responses for identical subthreshold synaptic barrages at each dendritic location (superimposed; 20-μm intervals centered between 35 μm [black traces] and 975 μm [gray traces] from the soma) for the indicated synaptic conductance types (RMP is −70 mV). Summed total synaptic currents are shown in ***B_2_***, bottom. Timings of synaptic activations are shown with semi-transparent black dots above the synaptic currents in ***B_2_***. While the spatiotemporal pattern is identical for all synaptic conductances, the number of synapses activated is adjusted for each conductance such that the maximal number of synaptic inputs that remain subthreshold across all dendritic locations is delivered. When occurring at locations where they are “just-subthreshold,” somatic EPSPs can recruit somatic and axonal voltage-gated sodium conductances, producing the small peaks visible on the largest somatic EPSPs. ***C***, Plots of the mean threshold numbers of synaptic activations necessary to initiate action potentials at different locations in the dendrite for synaptic inputs having AMPA-only (left; yellow), NMDA-only (middle; blue), or AMPA and NMDA (right; green) conductances, across seven different RMPs (−85 to −55 mV), as indicated by color depth. Mean ranges of synaptic thresholds measured across RMP (Δ_RMP_) or dendritic location (Δ_location_; ±SDs) and effect sizes (*d*, in units of *s*_AMPA-only_) of conductance on ranges (for NMDA-only and AMPA+NMDA inputs) are shown in the inset. ***D***, Plots of mean CVs (±SDs) calculated for the threshold number of synapses for each pattern of input (*n* = 10), across all locations within each RMPs (left), across all RMPs for each dendritic location (middle), and across all RMPs and dendritic locations (total CV; right) for inputs having AMPA-only (yellow), NMDA-only (blue), or both AMPA and NMDA (green) conductances in dendrites of the indicated lengths. Note that in the 1000-μm dendrite the CV for AMPA-only responses can be very large and off scale. The magnitudes of these large CVs are indicated next to their symbol (SD bars are to scale). The effect sizes (*d*) for changes in CV with AMPA+NMDA inputs (relative to AMPA-only CVs; expressed in units of *s*_AMPA-only_) are shown in green for each dendritic length.

Results are reported as mean ± SD. Because our simulations are deterministic, we know a priori that differences in synaptic responses are “real” (that is to say, we know they result from the changes in synaptic conductance coded into the simulation). To quantify the relative impact of synaptic conductance on the variability of synaptic integration (as measured by CV of synaptic thresholds) across RMPs and/or dendritic locations, we calculated the effect size (*d*) for each synaptic conductance as the mean difference in CV (relative to AMPA-only trials) normalized by the SD (*s*) of the AMPA-only trials, according to the following formula:

$$d=\frac{\left({\overset{¯}{x}}_{\mathrm{test}}-{\bar{x}}_{\mathrm{AMPA}-\mathrm{only}}\right)}{s_{\mathrm{AMPA}-\mathrm{only}}},$$

where ${\bar{x}}_{\mathrm{AMPA}-\mathrm{only}}$ and ${\bar{x}}_{\mathrm{test}}$ are the means of CVs of synaptic thresholds calculated across trials (e.g., across patterns of synaptic input, dendritic locations, and/or RMPs) for AMPA-only simulations and those with modified synaptic conductances, respectively, and _sAMPA-only_ is the SD for CVs in AMPA-only trials. Thus, *d* represents both the direction (by sign) and magnitude (in units of s_AMPA-only_) of differences in mean variability of synaptic thresholds. This generates a more conservative measure of effect size than other approaches (such as Cohen’s *d*) because the SD of synaptic thresholds for AMPA+NMDA trials was typically much smaller than for AMPA-only trials (see, for instance, the error bars in Figs. 2*D*, 6*C*). Quantifying effect size in this manner allows direct comparison of the relative impact of synaptic conductance on variability in neurons of different sizes and across different RMPs.

## Results

### Effect of synaptic conductance type on individual EPSPs

We first compared the relative efficacy of individual synapses consisting of AMPA-only, NMDA-only, or both (AMPA and NMDA) conductances in depolarizing the soma of a simplified and fully passive ball-and-stick model neuron (see Materials and Methods) with the RMP set to one of seven values (−85 to −55 mV; see Materials and Methods). As expected, given the cable properties of dendrites (Gulledge et al., 2005), the amplitude and kinetics of the resulting EPSPs depended on synaptic conductance type, RMP, and the dendritic location of synaptic input (Fig. 1*A*). NMDA conductances were larger at depolarized RMPs and/or in distal dendritic compartments (Fig. 1*B*), generating NMDA-mediated EPSPs that were largest under those conditions (Fig. 1*C*, light-blue plots). On the other hand, AMPA-only and AMPA+NMDA EPSPs had maximal amplitudes at the most hyperpolarized RMPs, where driving force was greatest (Fig. 1*C*, dark yellow and dark green plots). Across all dendritic locations and RMPs, somatic EPSP amplitudes averaged 0.48 ± 0.10, 0.11 ± 0.07, and 0.52 ± 0.09 mV for AMPA-only, NMDA-only, and AMPA+NMDA responses, respectively. Somatic EPSPs generated by AMPA+NMDA conductances were larger (effect size, d, of +0.45 relative to AMPA-only inputs) and less variable across dendritic locations and RMPs (CV = 0.18) than EPSPs generated by AMPA-only (CV = 0.22) or NMDA-only (CV = 0.65) inputs. Similarly, AMPA+NMDA somatic EPSPs had smaller absolute amplitude ranges across RMPs for each dendritic location (mean range of 0.14 ± 0.04 mV; *d* = −1.96 relative to AMPA-only inputs) relative to ranges for AMPA-only (0.21 ± 0.03 mV) or NMDA-only (0.21 ± 0.02 mV) EPSPs. While somatic EPSP amplitudes decremented with synaptic distance from the soma, amplitudes at the site of synaptic input became progressively larger at distal dendritic locations (Fig. 1*C*), and averaged 0.96 ± 0.68, 0.15 ± 0.11, and 1.00 ± 0.69 mV for AMPA-only, NMDA-only, and AMPA+NMDA inputs, respectively. AMPA+NMDA dendritic EPSPs were marginally larger than AMPA-only EPSPs (*d* = +0.06) and had slightly smaller absolute ranges across RMPs (*d* = −0.24). These data demonstrate that adding an NMDA conductance to individual AMPA-only EPSPs generates marginally larger EPSPs with slightly reduced variability across dendritic location and RMP.

### Impact of synaptic conductance on EPSP-spike coupling

Although the impact of the NMDA conductance on somatic EPSP variability was small for individual EPSPs (Fig. 1*A*, compare yellow and green traces), we reasoned that its impact might be amplified when dendrites experience barrages of excitatory synaptic input. To test this, we measured EPSP-spike coupling in ball-and-stick model neurons with dendrites of various lengths (200–1000 μm) and compared, across dendritic locations and RMPs, the threshold number of synaptic activations necessary for action potential initiation in the axon. Figure 2*A* shows results for the most proximal dendritic location in a model neuron with a 1000-μm dendrite with a RMP of −70 mV. In each model neuron, ten stochastic spatiotemporal patterns of synaptic input (“synaptic barrages”; see Materials and Methods) were delivered to progressively more distal 50-μm spans of the dendrite. For simulations involving AMPA-only, NMDA-only, or both synaptic conductances, the number of activated synapses within each barrage was iteratively increased at each dendritic location until an action potential was initiated. For each pattern of synaptic input, the threshold number of synapses necessary for action potential generation was determined for inputs occurring at different dendritic locations (incremented at 10-μm intervals) and across seven RMPs (−85 to −55 mV). As with single EPSPs, identical barrages of synaptic input generated ever-larger local dendritic depolarization at progressively more distal dendritic locations. Whereas the increase in local response amplitude with distance was linear when inputs involved AMPA conductances only, incorporation of the NMDA conductance, by itself or in combination with the AMPA conductance, allowed for supralinear distance-dependent increases in response amplitude and width, including long-lasting regenerative NMDA spikes, at distal dendritic locations. This can best be seen in Figure 2*B*, which shows somatic and local dendritic responses to identical patterns of subthreshold input (determined for each conductance type as the maximal number of synapses that remained subthreshold across all dendritic locations) in a neuron with an RMP of −70 mV.

**Figure 3.**
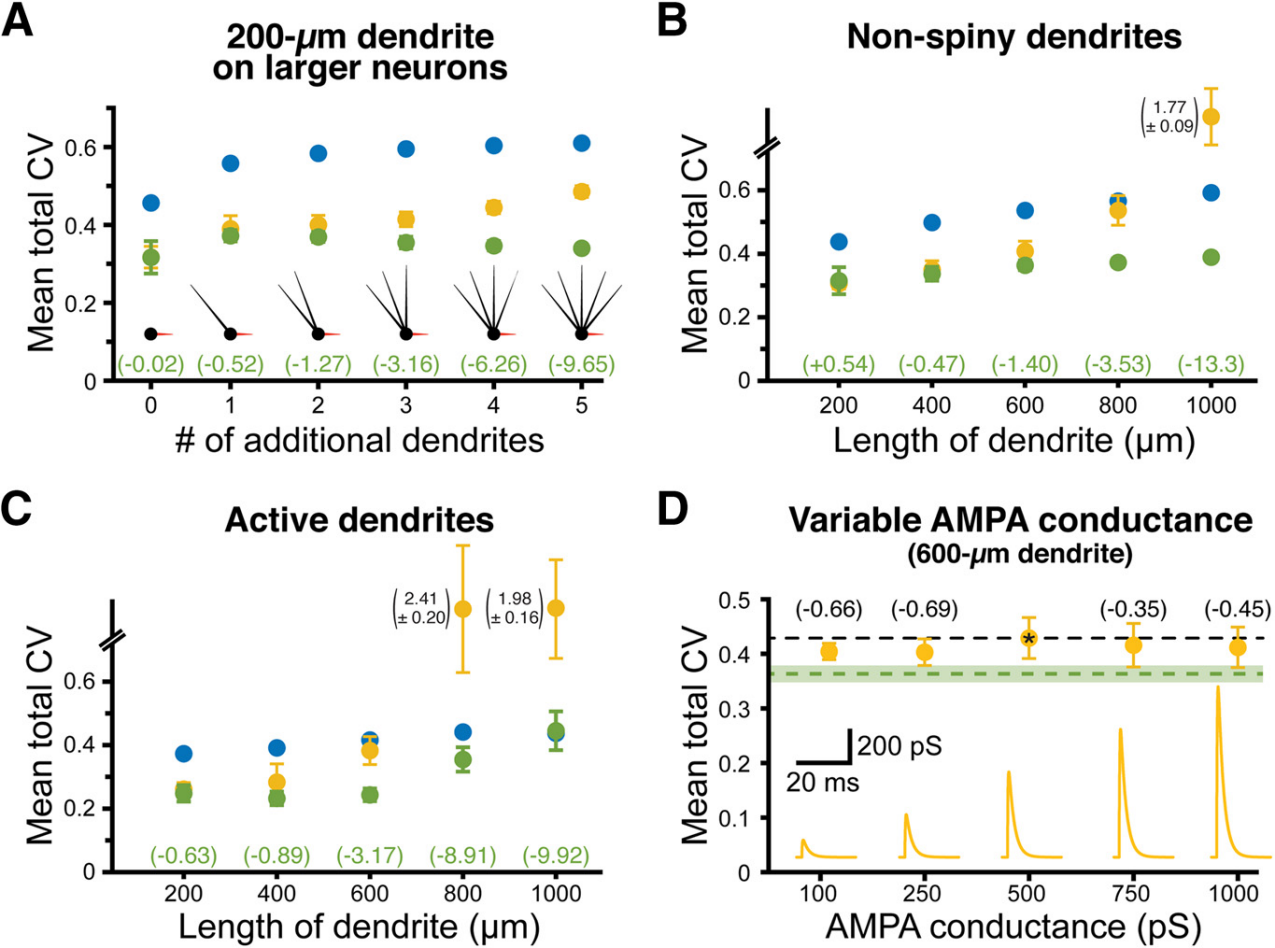
NMDA-dependent stabilization of EPSP-spike coupling is consistent across a range of model conditions and is not dependent on total synaptic conductance. ***A***, Total CVs (±SDs) for synaptic thresholds measured across all dendritic locations and RMPs for AMPA-only (yellow), NMDA-only (blue), and AMPA+NMDA (green) synaptic inputs to a 200-μm dendrite placed on neurons having 0–5 additional dendrites (600 μm each, as indicated in inset diagrams). Effect sizes for AMPA+NMDA inputs (relative to AMPA-only inputs) are listed in green (in units of *s*_AMPA-only_). The neuron with three additional dendrites is used in later figures and referred to as the “200-μm dendrite on a larger neuron.” ***B***, Total CVs (±SDs) for synaptic thresholds for AMPA-only (yellow), NMDA-only (blue), and AMPA+NMDA (green) inputs to non-spiny dendrites of the indicated lengths. Effect sizes for AMPA+NMDA CVs shown in green (units of *s*_AMPA-only_). ***C***, Total CVs (±SDs) for synaptic thresholds measured across all dendritic locations and RMPs for inputs onto spiny dendrites with active conductances (see Table 1). ***D***, Plot of mean total CVs for synaptic thresholds (±SDs) for AMPA-only EPSPs with the indicated peak conductance magnitudes in a 600-μm dendrite. Black dashed line indicates the mean CV of the standard 500 pS AMPA conductance (indicated by asterisk), while the green dashed line indicates mean total CV (with shaded SD) for AMPA (500 pS) + NMDA (1 nS) inputs in the same dendrite. Effect sizes of peak conductance manipulations, relative to the 500 pS AMPA input, are shown in black (in units of *s*_500pS_). Inset shows the AMPA conductances to scale.

For AMPA-only synapses, the mean threshold number of synaptic inputs increased with distance from the soma or with hyperpolarization of the RMP (Fig. 2*C*, left). This result reflects (1) distance-dependent voltage attenuation of summated EPSPs as they transfer to the soma; (2) smaller synaptic currents occurring when driving force is reduced during EPSPs in narrow, high-impedance dendrites (where local EPSP amplitudes are intrinsically larger and bring the membrane potential closer to the synaptic reversal potential); and (3) the necessity for greater depolarization to reach action potential threshold from hyperpolarized RMPs. On the other hand, when synapses contained only the NMDA conductance, the threshold number of synapses decreased with depolarization of the RMP but was fairly uniform across distance in the proximal dendrite before becoming lower at distal locations (Fig. 2*C*, middle). This shape resulted from distance-dependent voltage attenuation of somatic EPSPs combined with progressively larger synaptic currents at more distal, high-impedance dendritic locations where NMDA spikes of increasing amplitude and duration were observed (see Fig. 2*B*). Remarkably, when synapses contained both AMPA and NMDA conductances, synaptic thresholds for action potential generation were less sensitive to changes in RMP and less variable across distance than were thresholds for AMPA-only or NMDA-only synaptic inputs (Fig. 2*C*). While this reduced variability was evident from the mean ranges of synaptic thresholds (Fig. 2*C*), they were just as robust when threshold variability was normalized as CV. Whether measured across all dendritic locations (within any given RMP; Fig. 2*D*, left), across all RMPs (for any given dendritic location; Fig. 2*D*, middle), or across all dendritic locations and RMPs (“total CV”; Fig. 2*D*, right), and regardless of dendritic length, CVs of synaptic thresholds were always lowest when synapses contained both AMPA and NMDA conductances. Effect sizes were amplified in longer dendrites, likely reflecting their greater electrotonic diversity across input locations (see also Fig. 3).

Total CVs for synaptic thresholds, measured across all dendritic locations and RMPs for AMPA-only trials, ranged from a low of 0.32 ± 0.03 (in the 200-μm dendrite) to 2.07 ± 0.08 (in the 1000-μm dendrite; *n* = 10 patterns of synaptic input). When synapses incorporated both AMPA and NMDA conductances, CVs ranged from 0.32 ± 0.04 to 0.40 ± 0.01 (in the 200- and 1000-μm dendrites, respectively), with effect sizes (relative to AMPA-only trials) ranging from negligible (−0.02 *s*_AMPA-only_; see Materials and Methods) in the 200-μm dendrite, to enormous (−22 *s*_AMPA-only_) in the 1000-μm dendrite. Synaptic thresholds for NMDA-only conductances were always more variable than for inputs having both conductances, with total CVs ranging from 0.46 ± 0.01 to 0.61 ± 0.01 (in dendrites of 200 and 1000 μm, respectively) and effect sizes ranging from +3.28 *s*_AMPA-only_ (in the 600-μm dendrite) to −19.3 *s*_AMPA-only_ (in the 1000-μm dendrite; Fig. 2*D*).

To test whether the impact of dendritic length on synaptic threshold variability was reflective of electrotonic structure of the neuron, we measured synaptic thresholds in a 200-μm dendrite attached to larger neurons having one to five additional dendrites (each of 600 μm; Fig. 3*A*). Even as the measured dendrite remained static (at 200 μm), the effect size of combining AMPA and NMDA conductances on synaptic threshold variability was enhanced as neurons became progressively larger, ranging from almost nothing (−0.02 *s*_AMPA-only_) in the smallest neuron having no additional dendrites, to very large (−9.65 *s*_AMPA-only_) in the largest neuron with five additional dendrites (Fig. 3*A*). Similarly, making our normal ball-and-stick neurons electrotonically “tighter” by removing dendritic spines moderately reduced the impact of synaptic conductance on threshold variability (Fig. 3*B*; compare with the total CVs in Fig. 2*D*).

To test whether the impact of NMDA conductance depends on passive dendrites, in some simulations we added dendritic voltage-gated sodium and potassium conductances (see Table 1). Combining AMPA and NMDA conductances continued to reduce the variability of synaptic thresholds relative to AMPA-only inputs when dendrites contained active conductances (Fig. 3*C*).

Finally, to test whether the reduced variability in synaptic thresholds for AMPA+NMDA inputs was because of their larger peak conductance magnitudes, we repeated simulations using a range of AMPA-only conductances (100 pS to 1 nS) in a 600-μm dendrite. We found no consistent relationship between peak AMPA conductance and total CV of EPSP-spike coupling measured across all dendritic locations and RMPs (Fig. 3*D*; see also Fig. 7). This suggests that, rather than reflecting larger total synaptic conductance, the enhanced fidelity of EPSP-spike coupling observed with AMPA+NMDA inputs depends instead on properties intrinsic to the NMDA conductance.

### Synergistic interaction of voltage dependence and slow kinetics of the NMDA conductance

**Figure 4.**
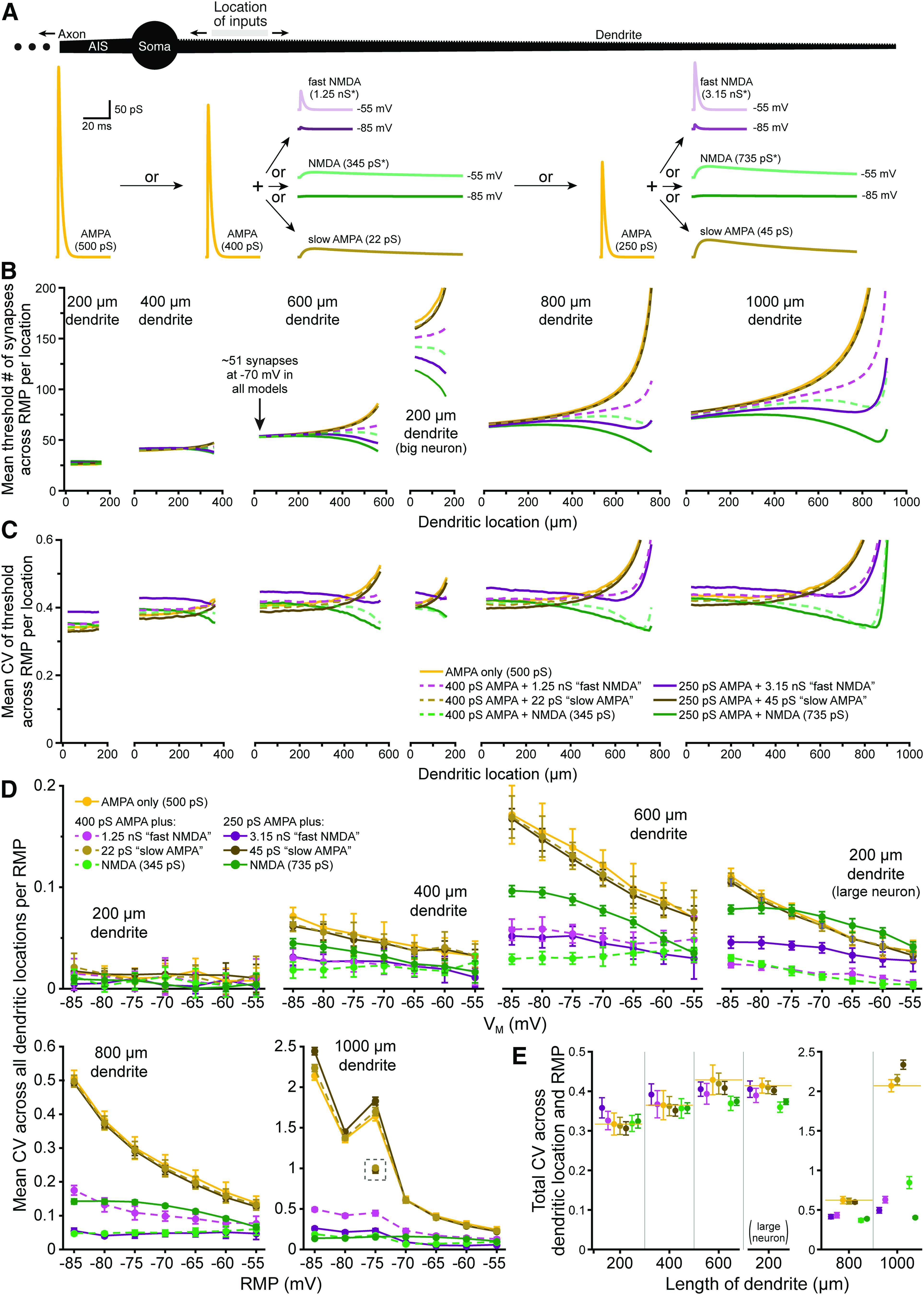
Differential impact of voltage dependence and kinetics on the fidelity of synaptic integration. ***A***, Diagram of a ball-and-stick neuron (top) receiving spatiotemporal patterns of synaptic input to 50-μm spans of dendrite to determine the number of synaptic inputs necessary for action potential generation. Traces below show the synaptic conductances tested: a 500 pS AMPA-only conductance, a 400 pS AMPA conductance “doped” with one of three additional conductances, including a fast-NMDA conductance (*nominally 1.25 nS maximal conductance, reaching 4.6 pS at −85 mV, and 49 pS at −55 mV, as shown), a slow-AMPA conductance with NMDA kinetics (22 pS), or a nominally 345 pS NMDA conductance (*reaching 1.3 and 13.6 pS at −85 and −55 mV, respectively), or a 250 pS AMPA conductance doped with relatively larger fast-NMDA, slow-AMPA, and NMDA conductances). All conductances are shown to scale and were titrated such that each generated a mean threshold of ∼51 synapses in the proximal end of the 600-μm dendrite at −70 mV (arrow in ***B***). ***B***, Plots of mean synaptic thresholds for inputs across all RMPs for each 50-μm span of dendrite (incremented at 10-μm intervals) for inputs having the indicated synaptic conductances in dendrites of the indicated lengths (*y*-axis cut off at 200 synapses; color coding as in panel ***C***). Note that we also tested a 200-μm dendrite attached to a large neuron having three additional 600-μm dendrites (middle plot; see also Fig. 3*A*). ***C***, Plots of mean CVs for thresholds calculated across seven RMPs (−85 to −55 mV) for each color-coded synaptic conductance at each dendritic location (i.e., measures of RMP-dependent variability of threshold for each given dendritic location) for the different length dendrites (as indicated in ***B***); *y*-axis cut off at 0.6. ***D***, Plots of mean CVs (±SDs) for synaptic thresholds for each color-coded conductance measured across all dendritic locations within each RMP (i.e., a measure of location-dependent variability in threshold for each RMP). For trials in the 1000-μm dendrite at −75 mV, the 500 pS AMPA and slow AMPA models hit very high thresholds (>3000) in their most distal measurable compartment, which generated large CVs at that RMP. CVs calculated for those conductances without the last dendritic location are plotted in the gray dashed box. ***E***, Plots of mean total CVs (±SDs) calculated across all dendritic locations and all RMPs for neurons with the indicated dendritic lengths and color-coded synaptic conductances.

The NMDA conductance differs from the AMPA conductance in two key ways: it is voltage dependent and has slower kinetics. To determine the relative impact of these properties on EPSP-spike coupling, we modified our simulations to measure variability in synaptic thresholds independently across location and RMP (Fig. 4). To do this, we started with our AMPA-only model (500 pS) and then replaced a portion of that conductance (100 or 250 pS) with either (1) the normal NMDA conductance; (2) a conductance having NMDA-like voltage dependence with AMPA-like kinetics (“fast-NMDA”); or (3) a conductance with NMDA-like kinetics but lacking voltage dependence (“slow-AMPA”). These added conductances were titrated so that each model exhibited a similar synaptic threshold for inputs to the proximal end of the 600-μm dendrite at −70 mV (∼51 synapses). The resulting synaptic conductances are shown to scale in Figure 4*A* following activation of individual synapses under voltage-clamp (voltage-dependent conductances shown for activations at −85 and −55 mV, as indicated). As in Figure 2, for each dendritic location (50-μm spans, incremented at 10-μm intervals), and RMP (from −85 to −55 mV, in 5-mV increments), we measured synaptic thresholds for action potential generation in response to each of ten randomized spatiotemporal patterns of synaptic input (Fig. 4*B*). We then calculated the CV of synaptic thresholds across RMPs for each dendritic location (Fig. 4*C*), across all dendritic locations at a given RMP (Fig. 4*D*), and across all dendritic locations and RMPs (total CV; Fig. 4*E*). This allowed us to dissociate the relative impacts of voltage dependance and conductance kinetics on the fidelity of EPSP-spike coupling. Adding back an AMPA conductance with slower kinetics (the brown plots from simulations including the slow-AMPA conductance in Fig. 4*C*) modestly reduced the CV of synaptic thresholds measured across RMP at a given dendritic location, especially in shorter dendrites or in proximal locations in longer dendrites. On the other hand, adding NMDA-like voltage dependence with fast, AMPA-like, kinetics (fast-NMDA; purple plots in Fig. 4*C*) increased the CV of synaptic thresholds measured across RMP at individual dendritic locations in small and/or proximal dendrites. Both of these effects were “dose dependent,” in that they were larger when they contributed proportionally more to the total synaptic conductance (Fig. 4*C*, compare dashed vs solid lines).

**Figure 5.**
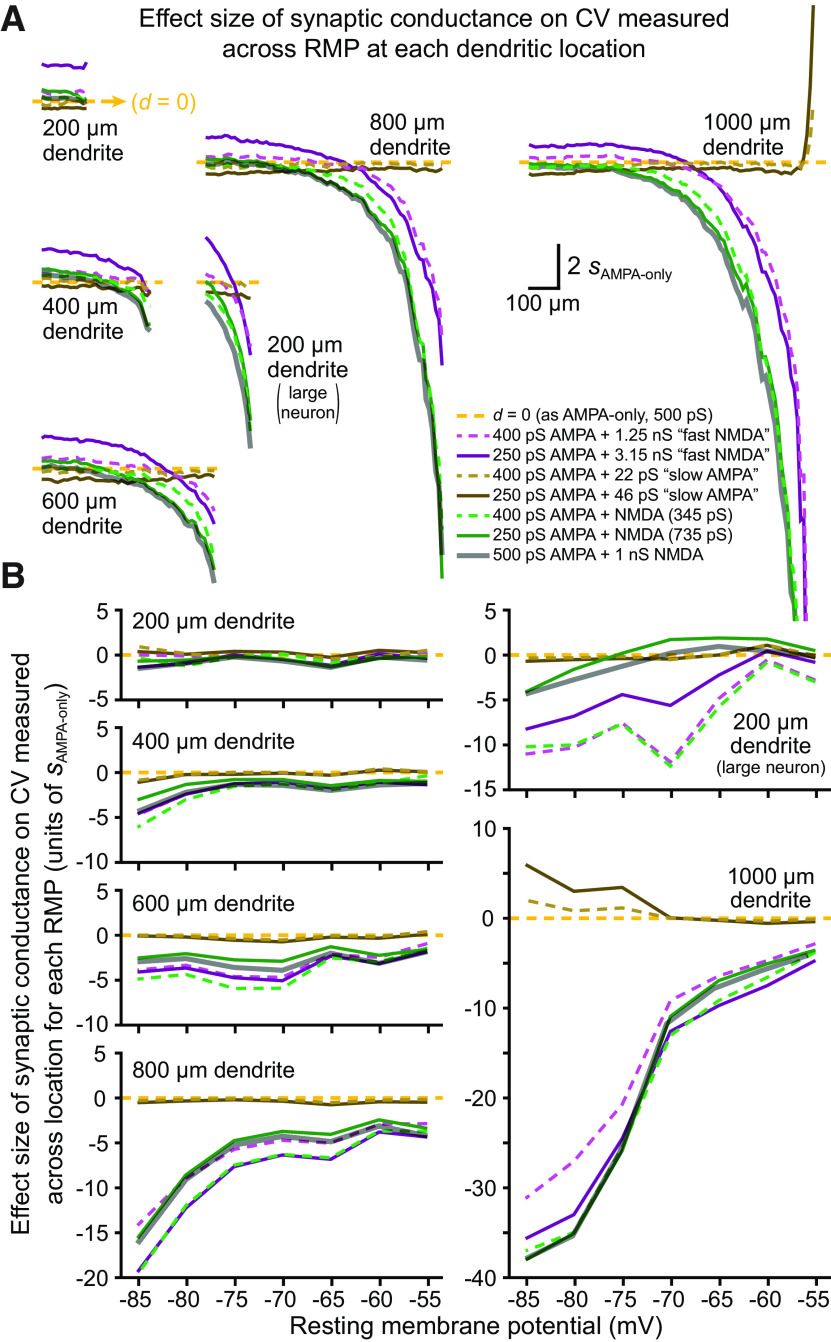
Effect sizes of synaptic conductance on variability of EPSP-spike coupling across RMP and dendritic location. ***A***, Plots of effect sizes of conductance-dependent changes in CV calculated across RMP for each dendritic location (in units of *s*_AMPA-only_; see Materials and Methods). Yellow dashed lines indicate *d**** ***=*** ***0 (i.e., identical to 500 pS AMPA-only trials), with effect sizes above that line reflecting larger-than-AMPA-only CVs, while values below the dashed-yellow lines indicate smaller-than-AMPA-only CVs. Data are from Figure 4*C*, and conductance types are similarly indicated by color. Effect sizes of our normal AMPA (500 pS) conductance combined with 1 nS NMDA (i.e., from Fig. 2*C*) are also shown with thick gray semi-transparent lines. Inputs with the fast-NMDA conductance tended to generate more variability in synaptic thresholds across RMP, especially in short or proximal dendrites, while inputs with slow kinetics exhibited smaller CVs relative to AMPA-only inputs. Combining voltage dependence with slow kinetics (e.g., green and gray NMDA traces) reduced variability in synaptic thresholds across RMP at all locations in most dendrites. Plots for the 1000-μm dendrite are truncated at −20 *s*_AMPA-only_. ***B***, Plots of effect sizes for changes in CV calculated across all dendritic locations within each RMP for data shown in Figure 4*D* (conductance types indicated by color, as in ***A***). Voltage-dependent conductances greatly reduced threshold variability across location within a given RMP (an effect magnified at hyperpolarized RMPs), whereas slow kinetics in the absence of voltage dependence had little effect.

When our normal NMDA conductance (titrated, as above) was added to these smaller AMPA conductances (Fig. 4*C*, green traces), CVs for synaptic thresholds measured across RMP were intermediate to those generated by fast-NMDA and slow-AMPA conductances, being only marginally larger than for AMPA-only inputs in short dendrites (or at proximal dendritic locations in longer dendrites) but becoming progressively lower at more distal dendritic locations. Overall, mean CVs of synaptic thresholds measured across RMP at all dendritic locations with titrated NMDA conductances were lower than for AMPA-only inputs in all dendrites longer than 200 μm, and in the 200-μm dendrite when attached to a larger neuron with three additional 600-μm dendrites (effect sizes ranging from −0.26 *s*_AMPA-only_ in the 400-μm dendrite to −1.91 *s*_AMPA-only_ in the 200-μm dendrite attached to a larger neuron). Effect sizes of conductance-dependent changes in synaptic threshold variability across RMP are plotted for each dendritic location in Figure 5*A*.

The impact of synaptic conductance on synaptic threshold variability was quite different when measured across all dendritic locations within a given RMP (Fig. 4*D*). For all dendrites >200 μm (and including in the 200-μm dendrite when attached to a larger neuron), the presence of the fast-NMDA conductance greatly lowered the CV of synaptic thresholds within any given RMP, while adding the slow-AMPA conductance had little impact on synaptic threshold variability across dendritic locations. The combination of voltage dependence and slow kinetics (i.e., our titrated standard NMDA conductance; green traces) generally lowed CV for synaptic thresholds measured across dendritic locations for a given RMP. For both fast-NMDA and regular NMDA conductances, the dose dependency was nonlinear, as the smaller dose typically generated the largest reductions in CV (relative to AMPA-only conductance; Fig. 4*D*). The effect sizes of these synaptic conductances on threshold variability across location within a given RMP (relative to 500 pS AMPA-only inputs) are shown in Figure 5*B*.

Finally, we measured total CV of synaptic thresholds across all dendritic locations and all RMPs for ten patterns of synaptic input (Fig. 4*E*). In smaller neurons (those with 200- and 400-μm dendrites), adding the fast-NMDA conductance (purple symbols) slightly increased CV relative to AMPA conductance alone. On the other hand, adding slow-AMPA kinetics (brown symbols) modestly reduced CV relative to AMPA-only inputs in most models. These effects were reversed in larger neurons such that the slow-AMPA conductance increased mean total CV in the 1000-μm dendrite, while the voltage-dependent fast-NMDA conductance reduced total CV for all dendrites longer than 400 μm, as well as in the 200-μm dendrite when it was attached to a larger neuron. This likely reflects the ability of the fast-NMDA conductance to effectively lower CV across distance in larger neurons by avoiding or delaying the exponential rise in synaptic thresholds observed for AMPA-only inputs (Fig. 4*C*), and that slower kinetics (i.e., slow-AMPA models) have reduced efficacy in lowering CV across RMPs at distal dendritic locations (Fig. 4*C*).

Combining voltage dependence with slow kinetics (i.e., the titrated normal NMDA conductance; Fig. 4*E*, green symbols) led to overall lower total CVs for synaptic thresholds in all dendrites longer than 200 μm, including in the 200-μm dendrite when attached to a larger neuron. Mean total CVs (across 10 synaptic input patterns) and effect sizes for conductance-dependent changes in total CV (relative to the 500 pS AMPA-only inputs) are shown in Table 2. In most dendrites, combining voltage dependence with slower kinetics had a synergistic effect in lowering total CV of synaptic thresholds to a greater extent than either manipulation on its own, often with “large” effect sizes (i.e., >1 *s*_AMPA-only_).

**Table 2:** Effect sizes for Figure 4*E*

Dendritic length (μm)	Conductance	gAMPA	Mean “total” CV	SD	Effect size (*d*)	gAMPA	Mean “total” CV	SD	Effect size (*d*)
200	AMPA-only ●	500	0.317	0.048					
	AMPA + fast NMDA	400 ●	0.326	0.023	**0.325**	250 ●	0.358	0.025	**1.469**
	AMPA + slow AMPA	400 ●	0.313	0.022	**–0.169**	250 ●	0.306	0.017	**–0.394**
	AMPA+NMDA	400 ●	0.319	0.020	**0.057**	250 ●	0.325	0.017	**0.263**
400	AMPA-only ●	500	0.365	0.280					
	AMPA + fast NMDA	400 ●	0.367	0.034	**0.060**	250 ●	0.392	0.027	**0.721**
	AMPA + slow AMPA	400 ●	0.363	0.024	**–0.071**	250 ●	0.352	0.014	**–0.371**
	AMPA+NMDA	400 ●	0.357	0.024	**–0.223**	250 ●	0.358	0.013	**–0.205**
600	AMPA-only ●	500	0.429	0.038					
	AMPA + fast NMDA	400 ●	0.394	0.026	**–0.940**	250 ●	0.406	0.018	**–0.623**
	AMPA + slow AMPA	400 ●	0.420	0.027	**–0.253**	250 ●	0.409	0.016	**–0.546**
	AMPA+NMDA	400 ●	0.370	0.018	**–1.578**	250 ●	0.375	0.010	**–1.450**
200 (on large neuron)	AMPA-only ●	500	0.414	0.019					
	AMPA + fast NMDA	400 ●	0.390	0.018	**–1.308**	250 ●	0.405	0.021	**–0.476**
	AMPA + slow AMPA	400 ●	0.410	0.016	**–0.222**	250 ●	0.402	0.010	**–0.666**
	AMPA+NMDA	400 ●	0.360	0.013	**–2.886**	250 ●	0.374	0.007	**–2.140**
800	AMPA-only ●	500	0.625	0.046					
	AMPA + fast NMDA	400 ●	0.434	0.035	**–4.131**	250 ●	0.415	0.027	**–4.535**
	AMPA + slow AMPA	400 ●	0.612	0.037	**–0.283**	250 ●	0.601	0.020	**–0.520**
	AMPA+NMDA	400 ●	0.369	0.023	**–5.537**	250 ●	0.389	0.010	**–5.104**
1000	AMPA-only ●	500	2.071	0.076					
	AMPA + fast NMDA	400 ●	0.630	0.041	**–18.964**	250 ●	0.495	0.036	**–20.737**
	AMPA + slow AMPA	400 ●	2.147	0.065	**1.002**	250 ●	2.337	0.058	**3.493**
	AMPA+NMDA	400 ●	0.847	0.077	**–16.111**	250 ●	0.405	0.010	**–21.928**

Listed are the mean “total” CVs, SDs, and effect sizes (relative to AMPA-only synapses) for synaptic thresholds calculated across all dendritic locations and all RMPs averaged from 10 patterns of synaptic input for each type of synaptic conductance (color coded to data in Fig. 4*E*). Effect sizes (d) indicate the normalized mean difference in CV relative to the CV for the 500 pS AMPA-only conductance, in units of sAMPA-only (see Materials and Methods). Sign indicates the direction of change [larger (+) or smaller (–) mean CVs than AMPA-only inputs]. Light shading (blue = smaller CVs, red = larger CVs) identifies manipulations generating mean changes in CV >0.5 SD, while darker shadings indicate mean changes >1.0 SD of the AMPA-only result. Note that AMPA+NMDA conductances generated consistently larger reductions in CV relative to manipulations that provided voltage dependence or slower kinetics alone. The large neuron with a 200-μm dendrite (tested) included three additional 600-μm dendrites (as in Fig. 3*A*).

To further compare the impact of synaptic conductance on the variability of EPSP-spike coupling, as measured across all RMPs (for a given dendritic location) or across all dendritic locations (for a given RMP), we plotted the effect sizes for our various synaptic conductances (relative to threshold variability observed for AMPA-only inputs) in Figure 5. This essentially transforms data in Figure 4*C*,*D* into measures of effect size (in units of *s*_AMPA-only_) to allow comparison of the differential impact of voltage dependence and slow kinetics on synaptic threshold variability across RMPs (Fig. 5*A*) or across all dendritic locations (Fig. 5*B*). Included in Figure 5 are results from our normal AMPA+NMDA conductance (500 pS AMPA + 1 nS NMDA; thick semi-transparent gray lines), which tended to track closely to the titrated NMDA conductances (green lines). As shown in Figure 5*A*, adding the fast-NMDA conductance induced a dose-dependent increase in the CVs for synaptic thresholds across RMPs in shorter dendrites and in the proximal two-thirds of longer dendrites (those ≥600 μm), with effect sizes for the larger dose of fast-NMDA (3.15 nS, paired with 250 pS AMPA) averaging +1.08 ± 0.26 *s*_AMPA-only_ (i.e., more variable thresholds across RMP relative to AMPA-only inputs) in shorter (200 and 400 μm) dendrite models and in the proximal two-thirds of longer dendrites. At distal locations in longer dendrites, effect sizes for NMDA-containing models became negative (i.e., reduced variability across RMP) and very large relative to AMPA-only inputs (Fig. 5). This occurred because adding a voltage-dependent component (regardless of kinetics) avoided, or at least delayed, the distance-dependent exponential climb in synaptic thresholds exhibited by AMPA-only inputs at distal dendritic locations (see Fig. 4*B*) and the corresponding increases in synaptic threshold variability across RMPs (see Fig. 4*C*). On the other hand, adding the larger dose of slow-AMPA conductance moderately lowered the variability of synaptic thresholds across RMPs for most proximal dendritic locations, with effect sizes averaging −0.46 ± 0.13 *s*_AMPA-only_ in all models >200 μm, including in the 200-μm dendrite when attached to a larger neuron (Fig. 5*A*).

Combining voltage dependence with slow kinetics (Figs. 4*C*, 5*A*, the green “NMDA” traces) revealed a location-dependent reduction in variability of synaptic thresholds measured across RMPs that was similar to that observed when adding the fast-NMDA conductance (Fig. 5*A*, compare green and purple traces). However, relative to fast NMDA trials, the slower kinetics of the normal NMDA conductance consistently led to lower CVs for synaptic thresholds at all dendritic locations in all model neurons. Mean effect sizes for all dendritic locations ranged from negligible (+0.06 *s*_AMPA-only_ in the 200-μm dendrite) to very large (−5.2 in the 1000-μm dendrite), and always became larger with increasing distance from the soma. As with the fast-NMDA conductance, the enormous effect sizes in distal dendrites reflects primarily the earlier (i.e., at more proximal dendritic locations) exponential increases in synaptic thresholds observed in AMPA-only models, rather than the more modest distance-dependent reduction in CV occurring in the presence of the NMDA conductance (Fig. 4*C*).

The impact of synaptic conductance on synaptic threshold variability measured across location within a given RMP is shown in Figure 5*B*. Regardless of kinetics, the addition of voltage dependence greatly reduced the CV of synaptic thresholds across location (Fig. 5*B*), and this effect was magnified in larger dendrites and at more hyperpolarized RMPs. The presence of slow kinetics in the absence of voltage dependence had little impact on synaptic threshold variability across dendritic location, except in the longest dendrite (1000 μm) at hyperpolarized RMPs, where variability increased relative to AMPA-only inputs.

Together, these results indicate that the impact of the NMDA conductance on the variability of synaptic thresholds across dendritic location and RMP reflects a synergistic interaction of multiple properties of the NMDA conductance, with its voltage dependence primarily enhancing fidelity across dendritic location within any given RMP, and its slower kinetics tending to enhance fidelity across RMP at a given dendritic location. These properties act together to greatly reduce the variability of EPSP-spike coupling across both RMP and dendritic location, relative to AMPA-only synaptic inputs.

### Synaptic integration in morphologically realistic neurons

**Figure 6.**
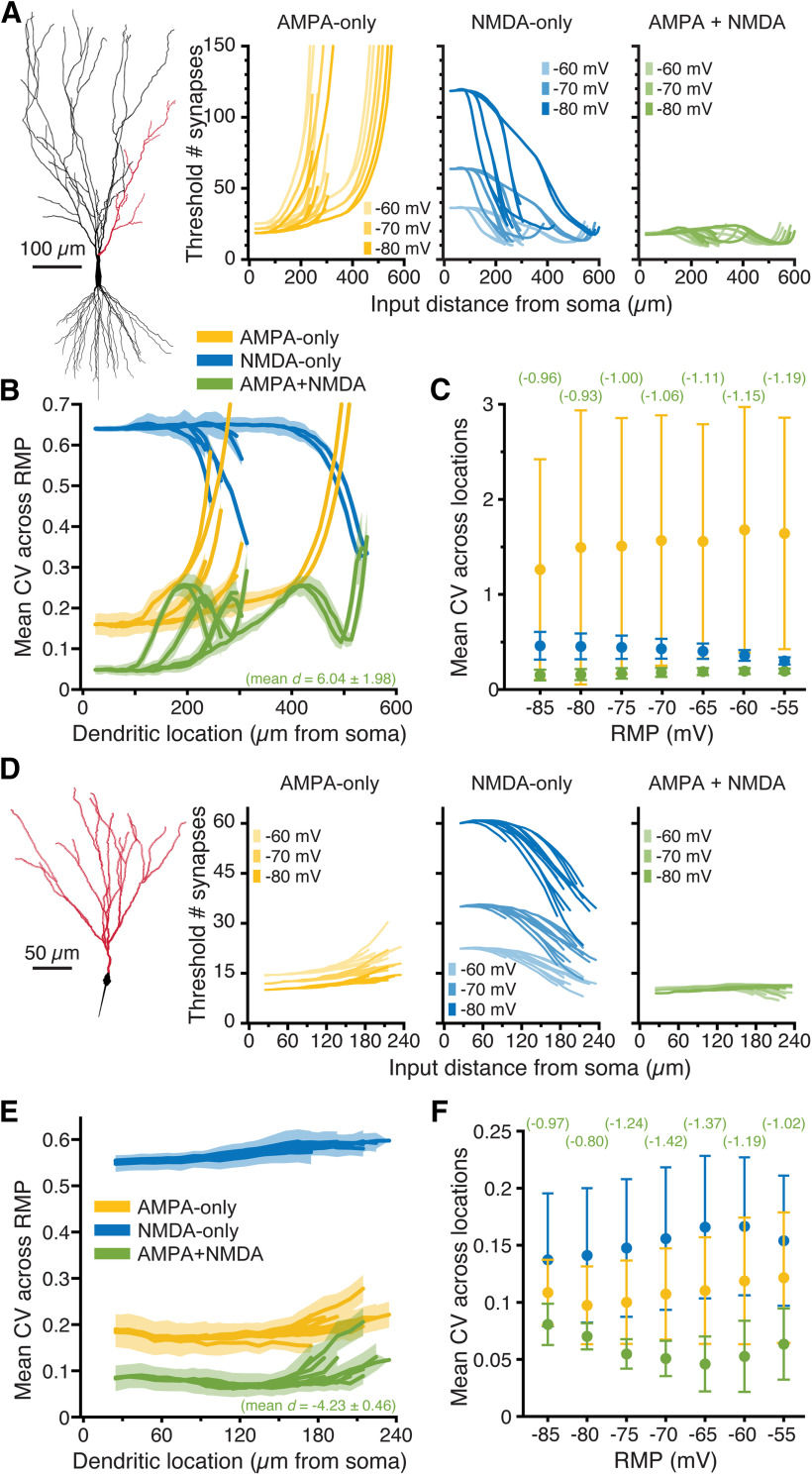
Impact of synaptic conductance on synaptic integration in realistic neuron morphologies. ***A***, Plots of the mean threshold number of synaptic activations necessary to depolarize the soma of a CA3 pyramidal neuron by 3 mV at progressively more distal 50-μm dendritic segments (along red dendrites in diagram at left) experiencing ten static-random spatiotemporal patterns of synaptic input. Colors (yellow, blue, and green) indicate simulations with AMPA, NMDA, and both AMPA and NMDA conductances, respectively, at three different RMPs (−60, −70, and −80 mV); *y*-axes are truncated at 150 synapses to show differences in proximal dendrites. ***B***, Plots of mean CVs for synaptic thresholds calculated across seven RMPs (−85 to −55 mV) for each dendritic location. Synaptic conductances indicated by color, with shaded regions indicating SDs. The mean effect size (*d*, in units of *s*_AMPA-only_) for CVs from AMPA-plus-NMDA simulations (relative to AMPA-only conductance) is shown in green at the bottom of the graph. ***C***, Comparisons of mean (±SD) CVs calculated for synaptic thresholds across dendritic locations within each of seven RMPs. Colors as in ***B***. Effect sizes for AMPA-plus-NMDA trials (vs AMPA-only inputs) are shown in green at top for each RMP (units of *s*_AMPA-only_). ***D***, Plots of the threshold number of synaptic activations necessary to drive a 5-mV somatic depolarization in a dentate granule neuron (red dendrites in diagram to left) at progressively more distal 50-μm segments experiencing ten expanding static-random spatiotemporal patterns of synaptic input. Colors as in ***B***. ***E***, Plots of mean CVs (with SD in shaded regions) of synaptic thresholds measured across seven RMPs for each dendritic location in the dentate granule neuron. Synaptic conductances indicated by color. ***F***, Comparisons of mean CV (±SD) for synaptic thresholds calculated across dendritic location within each of seven RMPs in the granule neuron. Effect sizes of AMPA-plus-NMDA inputs (relative to AMPA-only inputs) are indicated in green at top for each RMP (units of *s*_AMPA-only_).

The simulations above reveal that, in simplified neuron morphologies, the presence of both AMPA and NMDA conductances at synapses reduces location-dependent and RMP-dependent variability of somatic responses to spatially restricted barrages of synaptic input. To determine the impact of synaptic conductance on synaptic integration in realistic neuronal morphologies, we placed spinous synapses at 1-μm intervals across the dendritic trees of two reconstructed neurons: a relatively large CA3 pyramidal neuron (Fig. 6*A*), and a smaller dentate granule neuron (Fig. 6*D*). Because of their large dendritic arbors, it was not possible to drive action potential generation in these neurons with spatially restricted (50-μm) synaptic barrages, as even extreme depolarization of distal dendrites (e.g., clamping 50-μm spans to 0 mV) failed to depolarize the AIS to action potential threshold. Instead, we set arbitrary thresholds of somatic depolarization (3 mV above RMP for the CA3 neuron, and 5 mV above RMP for the smaller dentate granule neuron) and iteratively activated expanding stochastic patterns of synaptic input at different dendritic locations until somatic voltage thresholds were realized. RMPs were nominally set between −85 and −55 mV, in 5-mV increments, and synaptic barrages delivered to 50-μm spans along the dendritic branches indicated in red in Figures 6*A*,*D* (at 10-μm increments). In the CA3 neuron, the threshold number of AMPA-only inputs necessary to generate a 3-mV somatic depolarization increased with distance from the soma, or with depolarization of the RMP, as voltage attenuation and reduced driving force impaired synaptic depolarization of the soma (Fig. 6*A*, yellow plots). In contrast, when synapses contained only the NMDA conductance, the threshold number of synapses to depolarize the soma by 3 mV decreased with distance from the soma and/or with depolarization of the RMP, as distance-dependent increases in local input impedance, and/or depolarization of the RMP, enhanced the voltage-dependent NMDA conductance (Fig. 6*A*, blue plots). Combining both AMPA and NMDA conductances generated threshold numbers of synapses that were generally lower and more uniform across dendritic location and RMP (Fig. 6*A*, green plots). Whether measured across RMP (for a given dendritic location; Fig. 6*B*) or across dendritic location (within a given RMP; Fig. 6*C*), CVs were always less when AMPA and NMDA conductances were combined. Across RMPs, CVs of AMPA+NMDA inputs were reduced, on average, by 6.04 ± 1.98 *s*_AMPA-only_ (Fig. 6*B*), while effect sizes for reductions in CV across dendritic location (within a given RMP) ranged from −0.93 to −1.19, primarily because of large variability in the CVs of AMPA-only trials (Fig. 6*C*, compare the SDs of AMPA-only and AMPA+NMDA trials).

Across all dendritic locations and RMPs, total CVs in AMPA-only trials averaged 1.83 ± 0.10 across ten patterns of synaptic input, but were only 0.19 ± 0.01 in AMPA+NMDA trials, with an effect size of −16.6 *s*_AMPA-only_ for AMPA+NMDA inputs relative to AMPA-only trials. These results demonstrate that the combined presence of synaptic AMPA and NMDA conductances greatly reduces distance-dependent and RMP-dependent variability of synaptic integration (relative to AMPA-only inputs) in a realistic neuron morphology.

Similar results were observed in the smaller dentate granule neuron (Fig. 6*D*), where the threshold number of AMPA-mediated synaptic inputs increased with distance from the soma, or with depolarization of the RMP (Fig. 6*D*, yellow plots), because of distance-dependent voltage attenuation and reduced synaptic driving force, respectively. Conversely, for NMDA-mediated inputs, synaptic thresholds decreased with distance from the soma, or with depolarization of the RMP (Fig. 6*D*, blue plots). As was true in the CA3 neuron, the combined presence of synaptic AMPA and NMDA conductances in the dentate granule neuron minimized the impact of dendritic location and RMP on synaptic thresholds (Fig. 6*D*, green plots) and greatly reduced the CVs of thresholds calculated across RMPs for increasingly distant dendritic locations (Fig. 6*E*) or across dendritic location within a given RMP (Fig. 6*F*). Mean total CVs for the ten spatiotemporal input patterns, calculated across all dendritic locations and RMPs, were 0.19 ± 0.02, 0.52 ± 0.01, and 0.08 ± 0.01, for AMPA-only, NMDA-only, and AMPA-and-NMDA conductances, respectively, with an effect size of −5.18 *s*_AMPA-only_ for AMPA+NMDA inputs relative to AMPA-only inputs.

### Impact of AMPA-to-NMDA conductance ratios on synaptic integration

**Figure 7.**
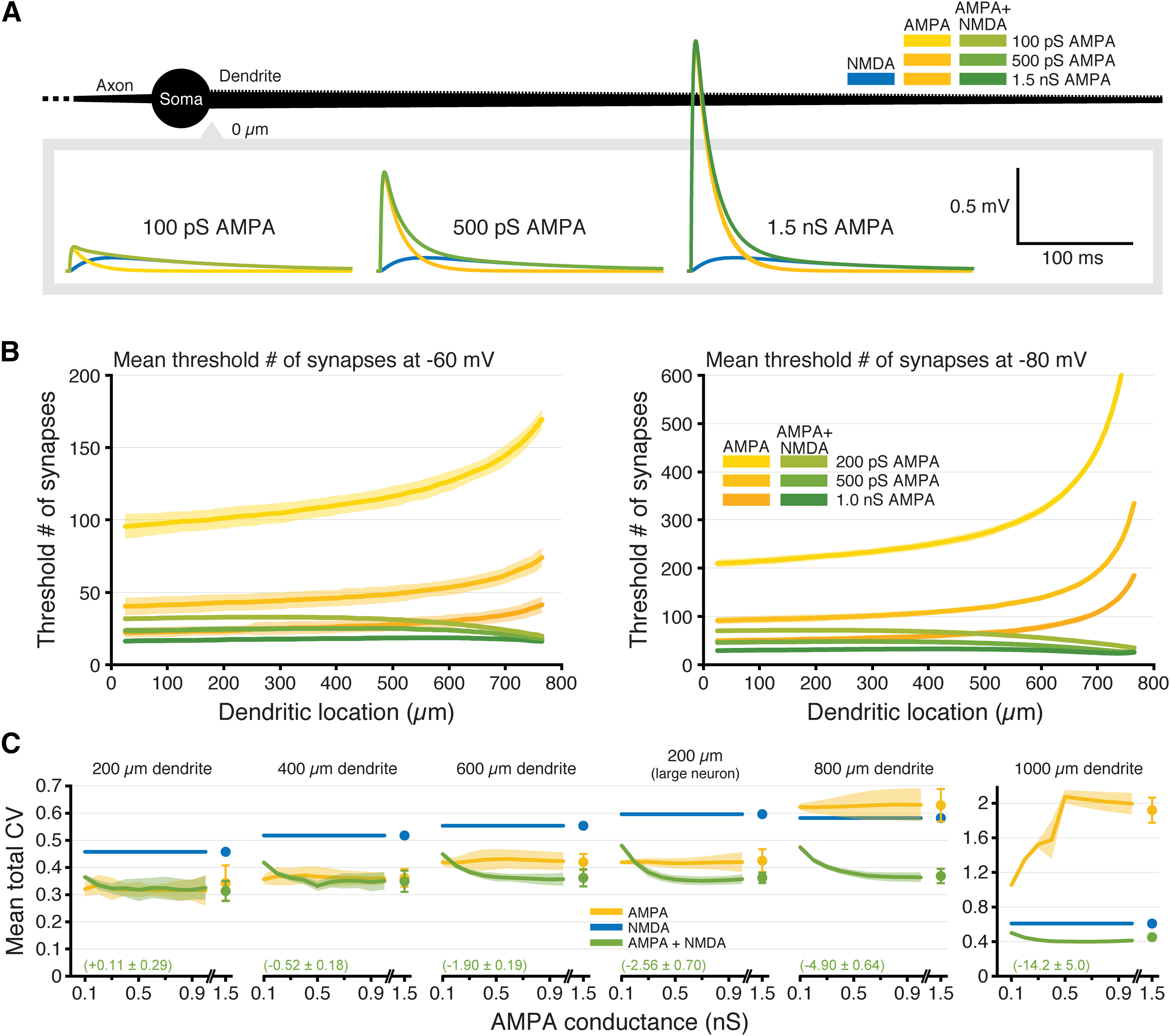
NMDA-dependent stabilization of EPSP-spike coupling occurs over a range of AMPA-to-NMDA conductance ratios. ***A***, Somatic EPSPs generated at −70 mV by proximal inputs having the indicated maximum AMPA conductances (maximal NMDA conductance held steady at 1 nS) in a ball-and-stick neuron (black; not drawn to scale). ***B***, Plots of the mean number of synaptic activations necessary to fire an action potential (±SDs; shaded regions) in an 800-μm-long ball-and-stick neuron resting at −60 mV (left) or −80 mV (right) with peak AMPA conductance set to 0.2, 0.5, or 1 nS, either alone (yellow) or together with a 1 nS NMDA conductance (green); *y*-axes limited to 200 (left) or 600 (right) synapses to show differences in proximal locations. ***C***, Plots of mean CVs for thresholds (±SDs; shaded regions) for each dendritic length (including the 200-μm dendrite on a large neuron with three additional 600-μm dendrites) for inputs having AMPA-only (yellow) or AMPA plus NMDA (green) conductances as a function of maximal AMPA conductance (0.1–1.5 nS). Mean effect sizes for AMPA+NMDA inputs (relative to AMPA-only inputs; ±SDs) calculated for all AMPA conductances ≥400 pS are shown in green. As a reference, the CV (±SD) for NMDA-only synapses is shown in blue. Note the *y*-axis scale change for the 1000-μm dendrite.

The simulations described above reveal that combining AMPA and NMDA conductances can increase the fidelity of synaptic integration, as measured at the soma and axon, across dendritic locations and/or RMPs. However, while the number of synaptic NMDA receptors is fairly uniform across most synapses (on the order of 10 receptors per synapse; Racca et al., 2000; Nimchinsky et al., 2004; Noguchi et al., 2005), synaptic AMPA conductances can range from zero (i.e., in “silent” synapses) to >1.5 nS (Beaulieu-Laroche and Harnett, 2018), and are dynamic in response to synaptic plasticity (for review, see Herring and Nicoll, 2016). Thus, the ratio of AMPA-to-NMDA conductance at synapses is variable. To determine the impact of AMPA-to-NMDA ratio on the fidelity of synaptic integration, we varied the maximal AMPA conductance from 0.1 to 1.5 nS while keeping the maximal NMDA conductance steady at 1 nS, and measured the minimum number of synaptic activations necessary to trigger an action potential in ball-and-stick neurons of various dendritic lengths (Fig. 7). Examples of individual synaptic responses generated by AMPA-only and AMPA+NMDA inputs containing different magnitudes of AMPA conductance are shown in Figure 7*A* (NMDA-only inputs are also shown for comparison). Regardless of AMPA conductance magnitude, the threshold number of synaptic inputs for action potential initiation increased sharply with distance from the soma for AMPA-only inputs, while thresholds for inputs containing both AMPA and NMDA conductances were relatively independent of distance (Fig. 7*B*). In long dendrites (>600 μm), the mean CV for the threshold number of inputs was lowest when both conductances were present, regardless of AMPA-to-NMDA ratio (Fig. 7*C*). In ball-and-stick model neurons with short dendrites, when AMPA conductances were small, adding an NMDA conductance did not significantly reduce threshold CVs, as the NMDA conductance became the dominant driver of somatic depolarization, and CVs converged toward those of NMDA-only inputs. This reflects the tight electrotonic structure of small model neurons, as adding NMDA conductance reduced the CV of EPSP-spike coupling across most AMPA-to-NMDA ratios in the 200-μm dendrite when it was attached to a larger neuron with three additional 600-μm dendrites (“large neuron” in Fig. 7*C*; compare also results from the short-but-branched dendritic arbor of the dentate granule neuron in Fig. 6*D–F*). For all AMPA conductances ≥400 pS, the mean effect size of adding NMDA conductance (relative to AMPA-only inputs) varied between negligible (*d* = +0.11 ± 0.29 *s*_AMPA-only_ in the 200-μm dendrite) to enormous (*d* = −15.2 ± 5.0 *s*_AMPA-only_ in the 1000-μm dendrite), with mean effect sizes always being more negative than −1.0 for dendrites ≥ 600 μm (including in the 200-μm dendrite attached to a larger neuron; Fig. 7*C*). Thus, combining AMPA and NMDA conductances enhances the fidelity of synaptic integration across a wide range of physiologically relevant AMPA-to-NMDA ratios.

## Discussion

**Figure 8.**
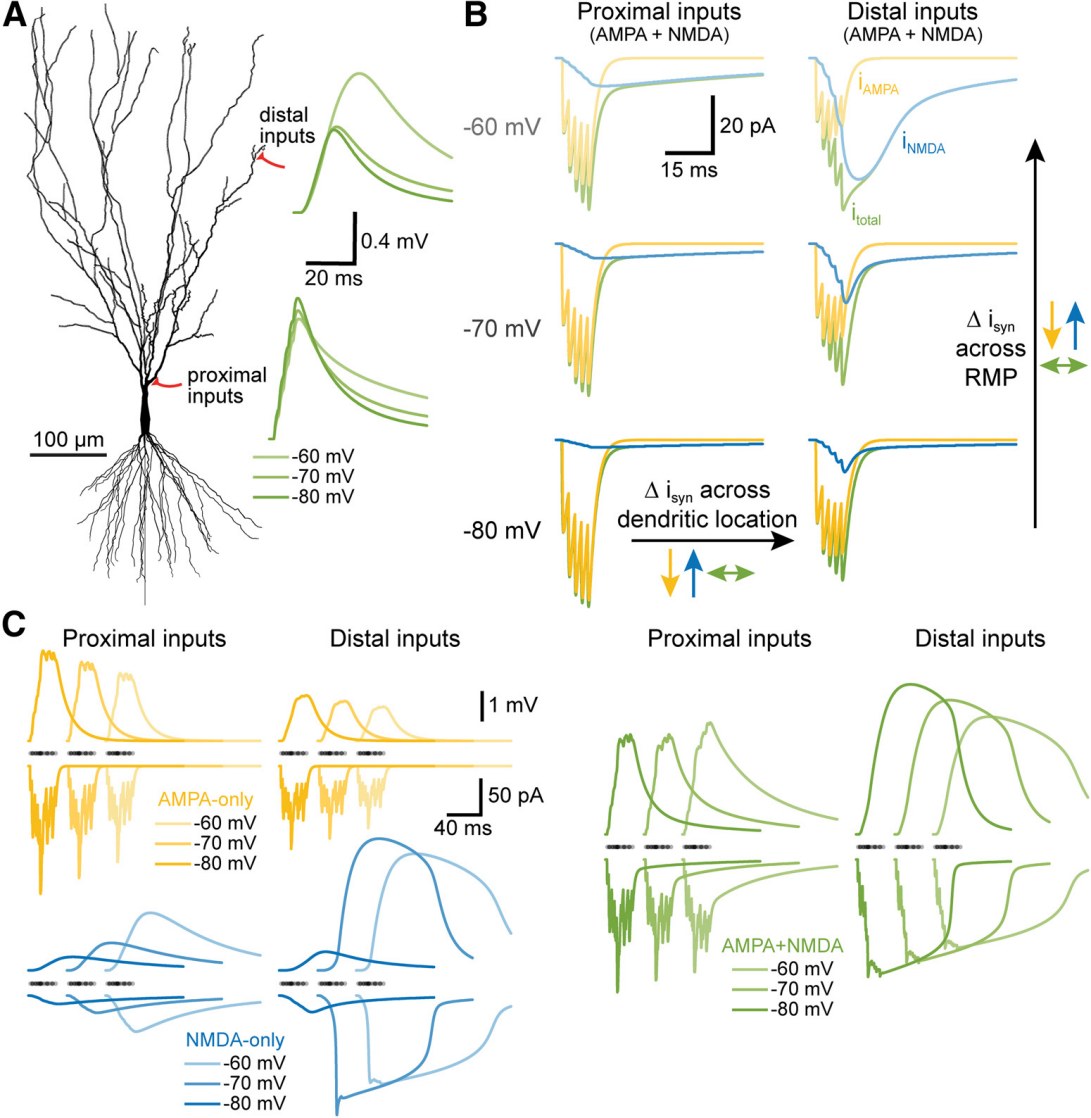
Summary of NMDA-dependent stabilization of synaptic integration across dendritic location and RMP. ***A***, Morphology of a CA3 neuron indicating the locations of proximal and distal synaptic inputs to the apical dendrite. Traces at right show the somatic responses to sequential activation of five AMPA+NMDA synapses at neighboring spines (at 2-ms intervals, distal to proximal) at three different RMPs (as indicated by color depth) for each input location (distal vs proximal) in a fully passive model. ***B***, Synaptic currents (i_syn_) generated by activation of the five proximal (left) or five distal (right) inputs at three RMPs. Total synaptic currents (i_total_) are shown in green, with AMPA (i_AMPA_; yellow) and NMDA (i_NMDA_; blue) components superimposed. Notice that AMPA currents are reduced (yellow arrows), while NMDA currents are enhanced (blue arrows), as inputs are moved from proximal to distal locations, or as RMP is depolarized from −80 to −60 mV. These opposing effects of location and RMP limit the variability of the total synaptic current across location and RMP (green arrows). ***C***, Similar to ***B***, but showing somatic voltage responses (upper traces) and summed synaptic currents (lower traces) for a barrage of 25 synaptic inputs with stochastic timings and spine locations for AMPA-only (yellow), NMDA-only (blue), or AMPA+NMDA (green) inputs. Timings of synapses indicated with semi-translucent dots above current traces. Note how AMPA-only currents and somatic EPSPs (yellow) get smaller with depolarization or distance from the soma, while NMDA-only currents and EPSPs (blue) get larger with distance and depolarization. Combining AMPA and NMDA conductance (green) leads to less variation in the amplitudes of both synaptic current and somatic EPSPs across RMPs and/or dendritic locations.

Synaptic integration, the process by which patterns of synaptic input are transduced into action potential initiation (also known as “EPSP-spike coupling”), is the core of neural computation. We tested the impact of two prominent and often coexpressed glutamate receptor subtypes, AMPA and NMDA receptors, on the fidelity of synaptic integration across dendritic location and neuronal state (i.e., initial membrane potential). Our results demonstrate that the kinetics and voltage dependence of the NMDA conductance act synergistically to counterbalance the impact of distance-dependent signal attenuation and the reduced synaptic driving forces occurring at depolarized membrane potentials, effectively increasing the fidelity of EPSP-spike coupling across both spatial and voltage domains (as summarized in Fig. 8). This intrinsic consequence of combining synaptic AMPA and NMDA receptors occurs over a broad range of neuron morphologies and AMPA-to-NMDA conductance ratios, and is independent of, but fully compatible with, its well characterized role in gating synaptic plasticity.

### Interaction of electrotonic structure and synaptic conductance

EPSPs are shaped by the electrotonic structure of the dendritic tree; in narrow distal dendrites, small surface areas limit local membrane conductance and capacitance, yielding greater input impedance and larger and faster local EPSPs in response to synaptic currents (Gulledge et al., 2005). This can reduce synaptic currents in distal dendrites, as greater local depolarization during EPSPs limits synaptic driving forces at the peak of the EPSP. At the same time, dendrites act as low-pass filters for EPSPs spreading from the site of synaptic input toward the soma. Thus, in the absence of voltage-dependent conductances, the somatic efficacy of synaptic input diminishes with distance from the soma, thereby requiring a greater number of synaptic inputs to reach action potential threshold. Our results with AMPA-only synaptic inputs are fully consistent with these well-described dendritic cable properties (Figs. 1, 2). NMDA conductances, on the other hand, generate larger synaptic currents in distal, high impedance dendritic compartments because of their enhanced conductance at depolarized membrane potentials (Gulledge et al., 2012; Lajeunesse et al., 2013). Our main result is that the combined presence of AMPA and NMDA receptors intrinsically lowers location-dependent and RMP-dependent variability in synaptic efficacy by boosting synaptic conductance preferentially in high-impedance dendritic compartments (Branco and Häusser, 2011; Gulledge et al., 2012) and/or when neurons are in depolarized states (Branco et al., 2010), conditions in which synaptic driving forces are diminished (see Figs. 2, 8). NMDA-dependent stabilization of synaptic integration was robust across neuron morphologies (Figs. 2, 3, 6) and over a wide range of AMPA-to-NMDA conductance ratios (Fig. 7), and persisted in the presence of active properties in dendrites (Fig. 3*C*) that can contribute to voltage-dependent amplification of inward current following synaptic activation (Magee and Johnston, 1995; Lipowsky et al., 1996; Branco et al., 2010).

By doping the AMPA conductance with voltage dependence (fast-NMDA) or slower kinetics (slow-AMPA; see Figs. 4, 5), we were able to measure the relative impact of these two distinct properties of the NMDA conductance. Adding voltage dependence to the AMPA conductance reduced the variability of synaptic thresholds for action potential generation when measured across dendritic location within a given RMP, especially in long dendrites, but generally increased variability of synaptic thresholds when measured across RMP at a given dendritic location. This reflects the gradual recruitment of the NMDA conductance as synaptic barrages occur in progressively more-distal higher-impedance locations, leading to larger distal synaptic currents (see Figs. 2*B*, 8) and therefore fewer synaptic activations to reach threshold. On the other hand, the increased variability of EPSP-spike coupling when barrages were initiated at different RMPs reflects the nonlinear amplification of synaptic responses with increased depolarization, leading to a greater range of synaptic thresholds across RMPs. The effects of the fast-NMDA conductance on synaptic threshold variability across RMPs is greater in proximal dendritic locations for two reasons. First, voltage-dependent amplification is limited in distal, high-impedance compartments because EPSPs (including NMDA-spikes) approach the synaptic equilibrium potential. Second, AMPA-only inputs exhibit larger and faster exponential increases in synaptic thresholds with distance from the soma (see Fig. 4*B*). Together, these processes likely explain why voltage dependence alone (i.e., the fast-NMDA conductance) generates relatively higher CVs across RMPs at proximal dendritic locations, but lower CVs across RMPs in distal dendrites.

The slower kinetics of NMDA receptors, on their own, reduced the variability of synaptic thresholds selectively across RMP at proximal dendritic locations by increasing the opportunity for temporal summation during barrages. At any point within the stochastic barrage, the next iterative input is more likely to summate to a suprathreshold response if EPSP decay is slow. Since the decay of EPSPs depends on the local time constant, which itself is location dependent (i.e., larger near the soma; compare, for instance, the decay rates of proximal vs distal EPSPs having identical kinetics in Fig. 1*A*), the impact of slower kinetics will be largest in proximal dendritic locations (see Figs. 4, 5). The overall impact of adding voltage-dependent and slower NMDA conductances to AMPA synapse is to greatly reduce the variability of somatic responses and EPSP-spike coupling across both dendritic location and membrane potential state (Figs. 2, 6).

### Impact of NMDA conductance on synaptic integration and network performance

Neurons employ a variety of mechanisms to combat location-dependent variability in synaptic efficacy. In the apical dendrites of CA1 pyramidal neurons, synaptic conductance is scaled in proportion to distance from the soma to minimize location-dependent variability of axonal drive (Magee and Cook, 2000; Nicholson et al., 2006; Shipman et al., 2013). Alternatively, dendrites may express dendritic voltage-gated sodium and calcium conductances that, with sufficient local depolarization, can generate dendritic action potentials that amplify somatic EPSPs (Magee et al., 1995; Magee and Johnston, 1995; Schiller et al., 1997; Golding and Spruston, 1998; Golding et al., 1999; Milojkovic et al., 2005). Similarly, synaptic NMDA receptors, when activated in sufficient numbers, generate self-sustaining plateau potentials (NMDA spikes) that lead to supralinear synaptic summation and larger somatic EPSPs (Schiller et al., 2000; Wei et al., 2001; Polsky et al., 2004; Nevian et al., 2007; Major et al., 2008; Larkum et al., 2009; Branco et al., 2010; Branco and Häusser, 2011; Harnett et al., 2012). These voltage-dependent synaptic and dendritic mechanisms act preferentially in distal dendritic compartments where input impedance is highest to facilitate synaptic depolarization of the soma and axon despite distance-dependent voltage-attenuation within the dendritic arbor. However, unlike action potentials, NMDA spikes are not all-or-none. Instead, they vary in amplitude and duration in proportion to the number of activated synapses, the local input impedance at the synapse, and the initial membrane potential (Major et al., 2008; Branco et al., 2010; Branco and Häusser, 2011; Gulledge et al., 2012; Farinella et al., 2014). Thus, although highly nonlinear across voltage, NMDA spikes are effectively “graded” in amplitude and duration across dendritic locations (see Fig. 2*B*), allowing the NMDA conductance to enhance synaptic current in proportion to electrotonic distance from the soma and axon. Similarly, by opposing the impact of reduced synaptic driving force on AMPA-mediated currents, voltage-dependent NMDA conductances can reduce the variability of total synaptic current during temporal integration of clustered excitatory inputs (Connelly et al., 2016; see also Fig. 8*C*).

Over the past several decades, there has been growing appreciation that, in addition to gating many forms of synaptic plasticity, NMDA receptors play an integral role in normal synaptic integration (for review, see Häusser and Mel, 2003; Antic et al., 2010; Major et al., 2013). In the distal apical tufts of layer 5 neurons, where high input impedance favors amplification of local EPSPs, NMDA activation boosts synaptic currents such that they can more reliably trigger calcium spikes at an electrically excitable zone at the base of the tuft (Larkum et al., 2009). Indeed, simulations by Larkum et al. (2009) found the threshold number of synapses necessary for initiating an apical trunk calcium spike to be relatively consistent across tuft locations when NMDA conductances were present, but that thresholds for AMPA-only inputs grew exponentially, and to non-physiological synaptic densities, with distance from the initiation zone in the apical dendrite. Thus, the impact of NMDA receptors on the fidelity of EPSP-spike coupling may not be limited to action potentials initiated at the axon, but likely applies more generally for spike initiation at any specialized trigger zone, so long as it is electrotonically downstream (e.g., in a larger compartment) from the site of synaptic input.

In conclusion, transduction of synaptic events into patterns of action potential output is the most fundamental neuronal task. It is a process influenced by the strength and kinetics of individual synaptic conductances, the spatiotemporal relationships among them, and the electrotonic properties of the neuron. Nonlinear amplification of synaptic input via NMDA spikes is proposed to increase the “computational power” of neurons (Wei et al., 2001; Poirazi et al., 2003; Branco and Häusser, 2011). Our results demonstrate that NMDA receptors, via their intrinsic kinetics and voltage dependence, also provide the advantage of “computational stability” by enhancing the fidelity of EPSP-spike coupling across dendritic domains and membrane potential states. While speculative, it is interesting to consider whether this consequence of NMDA receptor expression may have provided advantages to primitive nervous systems (e.g., in cnidaria; Anctil, 2009; Pierobon, 2012) independent of their role in associative plasticity, which may account for their ancient evolutionary origin in the common ancestor of all metazoans (Ramos-Vicente et al., 2018).

## Synthesis

Reviewing Editor: Niraj Desai, NINDS Decisions are customarily a result of the Reviewing Editor and the peer reviewers coming together and discussing their recommendations until a consensus is reached. When revisions are invited, a fact-based synthesis statement explaining their decision and outlining what is needed to prepare a revision will be listed below. The following reviewer(s) agreed to reveal their identity: Srdjan Antic.

Both reviewers and I agreed that this is a useful and thoughtful study of the impact of NMDARs on synaptic integration. However, we also agreed that you ought to clarify what is novel about your work. There have been many studies of this fundamental subject, and it is important to put your work in the context of the existing literature.

One of the reviewers wrote a particularly detailed and clear critique of the paper (reproduced below). All three of us agreed in whole or in part with the points they made (especially the first one about novelty and context). Please respond to it point by point.

********

CRITIQUE BEGINS HERE

********

This is an interesting study of basic membrane properties in dendrites of the central nervous system neurons. While dealing with well-known facts and receiving absolutely no help from novel experimental data, relying only on their imagination and cleverness, the authors were able to squeeze out some interesting observations about the behavior of a dendritic branch operating in the environment entirely based on AMPA and NMDA conductances. In this computer simulation study, the author’s best tools are two measures they developed: “CV of synaptic thresholds” and “Effect size (d)", where “d” is derived from “CV of synaptic thresholds” measurements in two subsequent conditions. These two numerical measures (“CV” and “d”) will help them generate enough graphical displays to account for one scientific paper.

There are some straight forward relationships that used to be straight-forward in the past, but are now packaged into a novelty. For example, because AMPA-mediated EPSPs are largest at hyperpolarized potentials (many publications), and NMDA-mediated EPSPs are largest at depolarized potentials (too many publications to cite), it is straightforward that AMPA+NMDA activation will average these extremes over the RMPs (VOLTAGE) and produce a family of EPSPs with smaller amplitude ranges than AMPA-only or NMDA-only.

Another straight forward example follows. Because AMPA-mediated EPSPs are largest when originate in proximal dendrite (tons of published work), and NMDA-mediated EPSPs are largest when originate in distal dendrite (both real data and model data were published), it is straightforward that AMPA+NMDA activation will average these extremes over DISTANCE and produce family of EPSPs with smaller amplitude ranges than AMPA-only or NMDA-only.

Nevertheless, I find certain educational value in the figures and statements of the current manuscript, and I think that some results are worth publishing.

I think that this manuscript should not be published if the authors fail to adequately modify the body of the manuscript, and resolve serious omissions in scientific presentation, plaguing the Results, Figures, Introduction, and Discussion. The manuscript deficiencies are listed below.

+++++++++++++++++++++++++++++++++++++++++++++++++++++++++++++++++++++++++++++++++++++

[1]

It is absolutely necessary to resolve the borders between the current study and the previous studies, which have already explained the importance of the AMPA-NMDA synergism, the impact of NMDA receptors on the spatial and voltage aspects of synaptic integration, and the impact of the AMPA-NMDA ratios.

To explain the novelty of their study, these authors wrote: “Here we report that the voltage-dependency and kinetics of NMDA receptors act synergistically to stabilize synaptic integration of excitatory postsynaptic potentials (EPSPs) across spatial and voltage domains.”

+++++++++++++++++++++++++++++++++++++++++++++++++++++++++++++++++++++++++++++++++++++

1.1. - Synergistically?

The first main finding of the authors is that AMPA and NMDA receptors act synergistically. I see a major overlap between this “novelty” and previously published facts. It was shown many years ago, in multiple publications, that AMPA receptor activation is essential for the function of NMDA receptors. In this sense, “the news” that AMPA and NMDA receptors act synergistically, is really not a news.

When researchers publish the impact of the AMPA-to-NMDA ratio on EPSP or synaptic integration, this is an example of synergistic action between two receptors.

It is the job of the authors, not the readers, to explicitly state that AMPA-NMDA synergism is well established (cite the original contributors) and then state exactly the author’s contribution to the AMPA-NMDA synergism here that warrants novelty?

+++++++++++++++++++++++++++++++++++++++++++++++++++++++++++++++++++++++++++++++++++++

1.2. - Spatial domains?

The second main finding of the authors is that AMPA and NMDA receptors act synergistically to stabilize synaptic integration of excitatory postsynaptic potentials (EPSPs) across spatial domains. I found major overlap between this “novelty” and previously published facts.

[1.2.a] Researchers have already established that NMDA receptors stabilize synaptic integration across spatial domains. In their influential article published in Science, Branco et al (2010) clearly stated: “... the NMDA-dependent mechanism produced strong sensitivity to the direction of synaptic input, even in short pyramidal cell dendrites, making it more general and sensitive to synaptic input, robust against timing jitter, ...”. Pertaining to distributions of synaptic inputs along individual dendritic branches (distribution in space), Branco and colleagues have already shown that NMDA receptors stabilize synaptic integration in the spatial domain.

[1.2.b] Researchers have already established that mechanisms involving NMDA receptors do not require spatially coincident input patterns. EPSP amplification by NMDA receptors occurs when synaptic inputs are either clustered onto individual dendrites, or when they are distributed throughout the dendritic tree (Connelly et al., 2016, Major et al., 2013). This means: “NMDA receptors stabilize synaptic integration across spatial domains, when synaptic inputs are distributed in space onto different dendritic branches of the same neuron”.

[1.2.c] Researchers have already established that “distal inputs are amplified with high gain and integrated over broader time windows”. This allows distal inputs to overcome their electrotonic disadvantage, and become surprisingly more effective than proximal inputs at influencing action potential output” (Branco and Hausser, 2010). This is very similar to the conclusion of the current paper EACH time the current paper deals with synaptic thresholds for action potential output.

[1.2.d] Analysis of the simulations revealed that the synaptic integration gradients can be explained by the interaction between active NMDA conductances and the progressive increase in dendritic input impedance toward the tip of the branch (Branco and Hausser, 2010). This is very similar to the conclusion of the current paper EACH time the current paper mentions that NMDA receptors improve voltage amplitudes in distal dendritic segment.

[1.2.e] Using uniform synaptic NMDA conductance density in the model, the researchers established that the synaptic integration gradients do not require any underlying gradients in the properties of the synapses or in the dendritic distribution of voltage-gated channels. The model could reproduce experimentally observed integration gradients using entirely uniform synaptic parameters and densities of voltage-gated NMDA channels (Branco and Hausser, 2010). This is very similar to uniform densities of voltage-gated NMDA channels in the present study.

[1.2.f] The amplitude of unitary EPSP at the soma of CA1 pyramidal cells is independent of synapse location across the entire range of afferent inputs (Cook and Magee, 2000). This is very similar to the results reported here.

It is the job of the authors, NOT THE JOB of the READERS, to explicitly state that NMDA receptors stabilize synaptic integration across spatial domain (cite the original contributors), and then state exactly the present author’s contribution to the NMDA role in the “space domain” that warrants novelty?

+++++++++++++++++++++++++++++++++++++++++++++++++++++++++++++++++++++++++++++++++++++

1.3. - AMPA/NMDA ratio?

[1.3.a] “asynchronous inputs can effectively interact over a wide time window to increase the local membrane depolarization and recruit more NMDAR conductance, thereby producing a broad window for synaptic integration at distal dendrites. This was reproduced with a range of NMDA:AMPA ratios (Figure S1E). This interaction between NMDA and AMPA receptors is sufficient to explain the increased NMDA component of single synapses at distal locations (Branco and Hausser, 2010).

[1.3.b] The impact of AMPA to NMDA receptor ratio on somatic voltage was discussed in Major et al., 2013.

[1.3.c] Desynchronizing the inputs is roughly equivalent to reducing the AMPA component: Time jitter has far less effect on the much slower NMDA component (Major et al., 2013).

It is the job of the authors, NOT THE JOB of the READERS, to explicitly state that AMPA:NMDA ratio was thoroughly explored in the context of synaptic integration (cite the original contributors), and then state exactly the present author’s contribution to this topic that warrants novelty? How is the present author’s AMPA/NMDA ratio conclusion different from the conclusions stated in the published literature studying efficacy of synaptic integration in silico?

+++++++++++++++++++++++++++++++++++++++++++++++++++++++++++++++++++++++++++++++++++++

1.4. - Identical outline and similar result

"Figure 1” is conceptually identical, and result-wise similar, to:

Figure 4b, yellow, Magee, 2000.

Figure 1 of Gulledge et al., 2005.

Figure 1F of Major et al., 2013.

This overlap should be mentioned in the paper, and the present author’s novel contribution should be discussed.

+++++++++++++++++++++++++++++++++++++++++++++++++++++++++++++++++++++++++++++++++++++

1.5. - NMDA spike width in distal dendrite

"Whereas the increase in local response amplitude with distance was linear when inputs involved AMPA conductances only, incorporation of the NMDA conductance, by itself or in combination with the AMPA conductance, allowed for supralinear distance-dependent increases in response amplitude and width, including long lasting regenerative NMDA spikes, at distal dendritic locations (Figure 2B)”.

Distance-dependent increase in NMDA-mediated response width (duration of NMDA spike) was demonstrated by Gao et al., 2019 (BioRxiv), their figure 5. The authors should state their improvement over Gao et al., 2019.

+++++++++++++++++++++++++++++++++++++++++++++++++++++++++++++++++++++++++++++++++++++

[2] - Cardinal error in modeling soma and axon

The authors discuss AMPA and NMDA receptor activation in the context of cortical pyramidal neurons (neocortical pyramidal or hippocampal pyramidal) - 99% of references is on pyramidal neurons. Consequently, they use a model of a hippocampal CA3 pyramidal neuron. However, in the ball-and-stick model (Fig. 2A), and very likely in other figures as well, the authors use a highly inappropriate mechanism for action potential generation, with pronounced fast afterhyperpolarization, characteristic of hippocampal interneurons, not pyramids. Should the authors wish to inform themselves of the natural AP waveforms in the somata of hippocampal pyramids, they should examine Raus Balind et al., 2019.

Anyhow, with super-strong FAST afterhyperpolarization occurring in the axon, soma and proximal dendrites, the authors have unnaturally altered the waveforms of EPSPs in dendrite. For this reason, the computer exercises shown in figures must be revised (repeated) and re-analyzed to include axon mechanisms suitable to pyramidal neurons, producing somatic voltage waveforms that resemble those in pyramidal somata responding to dendritic suprathreshold excitation (Raus Balind et al., 2019).

+++++++++++++++++++++++++++++++++++++++++++++++++++++++++++++++++++++++++++++++++++++

[3] - Delete Figure 5.

Figure 5, and all accompanying text, should be deleted from the manuscript. It takes several hours to understand figures 4 and 5.

Figure 5 contributes little to the impact of the paper.

Fig. 5 is too complex and ineligible - the authors are strongly advised to remove it.

+++++++++++++++++++++++++++++++++++++++++++++++++++++++++++++++++++++++++++++++++++++

[4] - Simplify Fig. 4

Figure 4 is too dense and ineligible.

The middle panel of 4A should be DELETED. The medium size changes, contributed nothing to the manuscript. Deletion of the medium size panel in 4A will eliminate medium-size lines in 4B, 4C, 4D, to alleviate the clutter.

The strange, tumor-like graph “200 um dendrite (big neuron)", should be deleted from Fig. 4B. It severely violates the logic of presentation, composition of the figure panel, and compromises reader’s understanding of the figure.

The “200 um dendrite (big neuron)” graph insert should be deleted from Fig. 4C.

The “200 um dendrite (big neuron)” graph insert should be deleted from Fig. 4D and 4E,

to alleviate clutter and help important messages come forward.

+++++++++++++++++++++++++++++++++++++++++++++++++++++++++++++++++++++++++++++++++++++

[5] - The authors write in a cryptic style, leaving out the important details and explanations

The following text is an example of a highly problematic writing style frequently employed in the Results.

>> “To test whether the impact of dendritic length on synaptic threshold variability was reflective of electrotonic structure of the neuron, we measured synaptic thresholds in a 200-μm dendrite attached to larger neurons having 1-to-5 additional dendrites (600 μm each; Figure 3A). Even as the measured dendrite remained static (at 200 μm), the effect size of combining AMPA and NMDA conductances on synaptic threshold variability was enhanced as neurons became progressively larger, ranging from almost nothing (-0.02 sAMPA-only) in the smallest neuron having no additional dendrites, to very large (-9.65 sAMPA-only) in the largest neuron with five additional dendrites (Figure 3A). Similarly, making our normal ball-and-stick neurons electrotonically “tighter” by removing dendritic spines moderately reduced the impact of synaptic conductance on threshold variability (Figure 3B)”.

The following text is the same text repaired (with some bad English, because the reviewer is not a native speaker).

>> “From experiments shown in Fig. 2, we learned that the synaptic thresholds become more stable (less variable) when synapses incorporated both AMPA and NMDA conductances, as opposed to AMPA-only and NMDA-only synapses. The mean difference in CV (relative to AMPA-only trials) normalized by the standard deviation (s) of the AMPA-only trials, the so called “effect size (d)” (see Methods), here serves as a measure of the improvement in stability. In this respect, if AMPA+NMDA synapses improve the stability of synaptic thresholds over the AMPA-only, or NMDA-only synapses, the effect size “d” becomes negative. Also, “d” is proportional to the magnitude of improvement; a greater negative “d” means greater reduction in synaptic threshold variability caused by the AMPA+NMDA synapse design. To test the impact of neuronal morphology on the observed stabilizing effects of AMPA+NMDA synapse, we measured synaptic thresholds in a 200-μm dendrite attached to neurons having 1-to-5 additional dendrites (600 μm each; Figure 3A). We found that adding dendritic branches to synthetic neurons improved the effect of the AMPA+NMDA synapses on the stability of the synaptic threshold. Even as the measured dendrite remained static (at 200 μm), the effect size (d) was increased as neurons became progressively larger, ranging from almost nothing (d = -0.02 units) in the smallest neuron having no additional dendrites, to very large (d = -9.65 units) in the largest neuron with five additional dendrites (Figure 3A). These data indicate that the stabilizing effect of AMPA+NMDA synapses on synaptic thresholds is even greater in neurons with more elaborate dendritic trees (more branches). Similarly, making our normal ball-and-stick neurons electrotonically “tighter” by removing dendritic spines moderately reduced the impact of synaptic conductance on threshold variability (Figure 3B)”.

The authors should employ similar “repairs” throughout the Results, everywhere. This should be done by adding explanations and helping readers of all levels. In the current form, this manuscript is written for the lab members, who have had multiple prior exposures to these experimental outlines, and who use the same jargon.

George Gopen & Judith Swan (2018) explained my point #[5]: “The fundamental purpose of scientific discourse is not the mere presentation of information and thought, but rather its actual communication. It does not matter how pleased an author might be to have converted all the right data into sentences and paragraphs; it matters only whether a large majority of the reading audience accurately perceives what the author had in mind”.

+++++++++++++++++++++++++++++++++++++++++++++++++++++++++++++++++++++++++++++++++++++

[6] - invention of new biophysical units

”... ranging from almost nothing (-0.02 sAMPA-only) in the smallest neuron having no additional dendrites, to very large (-251 9.65 sAMPA-only) in “

Here, the authors should use the syntax “units”. Description of the sAMPA-only unit, and sAMPA-only calculation, should be performed in the Methods (as they have already done).

+++++++++++++++++++++++++++++++++++++++++++++++++++++++++++++++++++++++++++++++++++++

[7] - Origin of peak in somatic traces?

In Figure 2B, some of the large somatic voltage waveforms (NMDA-only and NMDA+AMPA) have small (5 ms-wide) peaks on the plateau phase. The dendritic waveforms, which are simultaneously obtained with somatic traces, are without that same peak. In the manuscript, please explain the origin of the small 5-ms wide peak observed in the cell body?

+++++++++++++++++++++++++++++++++++++++++++++++++++++++++++++++++++++++++++++++++++++

[8] - Error in Figure 2

"B, Somatic (B1) and dendritic (B2, top) voltage responses for identical subthreshold synaptic barrages at each dendritic location ...”

Semi-transparent black dots above the synaptic currents in Fig. 2-B2 indicate that the temporal structure of inputs was different for AMPA-only versus 2 other conditions. The temporal structure of synaptic barrages were not identical. Therefore, it is impossible for readers to compare three conditions under the same input paradigm.

+++++++++++++++++++++++++++++++++++++++++++++++++++++++++++++++++++++++++++++++++++++

[9] - Error in Figure 2

Ten stochastic spatiotemporal patterns of synaptic input (“synaptic barrages,” see Methods) were delivered to progressively more distal 50-μm-spans of the dendrite.

Readers can see multiple superimposed traces in Figure 2B corresponding to different segments of dendrite. The display is complete for “NMDA-only” and “AMPA+NMDA”. The display is incomplete for “AMPA-only", as multiple traces from multiple dendritic segments are missing.

We cannot understand why progressive distances were NOT modelled / displayed for “AMPA-only”? There is no explanation in the manuscript, and no explanation in figure legend.

+++++++++++++++++++++++++++++++++++++++++++++++++++++++++++++++++++++++++++++++++++++

[10] - Error in Figure 2

"For simulations involving AMPA only, NMDA-only, or both synaptic conductances, the number of activated synapses within each barrage was iteratively increased until an action potential was initiated”.

Unfortunately, we do not see AP initiation in Figure 2B1 or 2B2. We do not understand this discrepancy between the body of the manuscript and the figure.

"For each pattern of synaptic input, the threshold number of synapses necessary for action potential generation was determined for inputs occurring at different dendritic locations (incremented at 10-μm intervals) and across seven RMPs (-85 to -55 mV)”.

We do not see seven RMPs in Fig. 2B. The entire section of the manuscript devoted to Fig. 2B Results, is one big mistake in scientific communication, with multiple discrepancies between the body of the manuscript and the figure.

"axon (Figure 2A). Ten stochastic spatiotemporal patterns of synaptic input (“synaptic barrages,” see Methods) were delivered to progressively more distal 50-μm-spans of the dendrite. For simulations involving AMPA only, NMDA-only, or both synaptic conductances, the number of activated synapses within each barrage was iteratively increased until an action potential was initiated. For each pattern of synaptic input, the threshold number of synapses necessary for action potential generation was determined for inputs occurring at different dendritic locations (incremented at 10-μm intervals) and across seven RMPs (-85 to -55 mV). As with single EPSPs, identical barrages of synaptic input generated ever-larger local NMDA receptors stabilize synaptic integration, page 8 dendritic depolarization at progressively more distal dendritic locations. Whereas the increase in local response amplitude with distance was linear when inputs involved AMPA conductances only, incorporation of the NMDA conductance, by itself or in combination with the AMPA conductance, allowed for supralinear distance-dependent increases in response amplitude and width, including long lasting regenerative NMDA spikes, at distal dendritic locations (Figure 2B)”.

+++++++++++++++++++++++++++++++++++++++++++++++++++++++++++++++++++++++++++++++++++++

[11] - Communication breakdown

In Figure 2D, we learned that the authors have employed sticks of different length, while in Fig. 2ABC we were led to believe that all simulations were performed on the same stick length of 1,000 um.

At what point the authors change their strategy?

When they discuss synaptic inputs delivered at 600 um distance from the cell body in Fig. 2ABC, does that mean that the total length of the stick in that model exercise was 600 um or 1,000 um? The authors need to clarify their procedures.

+++++++++++++++++++++++++++++++++++++++++++++++++++++++++++++++++++++++++++++++++++++

[12] I = g (Eq - Vm)

"This shape resulted from distance-dependent voltage attenuation of somatic EPSPs combined with progressively larger synaptic currents at more distal, high-impedance dendritic locations”.

Since the authors promised to deliver IDENTICAL synaptic drives along the length of dendrite at the same voltage, then the synaptic currents cannot possibly be different in repeated trials. Could you please explain your finding that despite identical synaptic conductance (synaptic input), identical equilibrium potential, and identical membrane potential, the synaptic current “I_syn” undergoes changes? Did the authors mean to say: “progressively larger synaptic potentials at more distal location”?

+++++++++++++++++++++++++++++++++++++++++++++++++++++++++++++++++++++++++++++++++++++

[13] - in figure 6c?

"while effect sizes for reductions in CV across dendritic location (within a given RMP) ranged from -0.93 to -1.19, primarily because of large variability in the CVs of AMPA-only trials (compare the standard deviations of AMPA-only and AMPA+NMDA trials in Figure 6B)”.

This sentence is perhaps erroneously referencing Figure 6B, when instead it should point to Figure 6C?

+++++++++++++++++++++++++++++++++++++++++++++++++++++++++++++++++++++++++++++++++++++

[14] - Figure 7A is AWOL

Figure 7A was never mentioned in the manuscript, and we, the readers, feel uncomfortable. Did we miss something important?

+++++++++++++++++++++++++++++++++++++++++++++++++++++++++++++++++++++++++++++++++++++

[15] - I do not see what author see

In ball-and-stick model neurons with short dendrites, when AMPA conductances were small, adding an NMDA conductance did not significantly reduce threshold CVs, as the NMDA conductance became the dominant driver of somatic depolarization, and CVs converged toward those of NMDA-only inputs.

In Fig. 7C, I do not see AMPA+NMDA CVs converging to NMDA-only CVs.

The authors need to either change the figure, or supplement the figure with labeled arrows, or change verbal interpretation, or all three.

+++++++++++++++++++++++++++++++++++++++++++++++++++++++++++++++++++++++++++++++++++++

[16] - Physical units of “Fidelity”?

"Thus, the voltage-dependence of the NMDA conductance enhances the fidelity of synaptic integration across a wide range of physiologically relevant AMPA-to-NMDA ratios”.

In their modeling exercises, the authors have produced some numerical values for:

[A] Threshold # of synaptic inputs,

[B] CV of Threshold # of synaptic inputs,

[C] Effect size (d).

Scientific measurements of FIDELITY were not provided. Please define here “Fidelity” in physical terms, or eliminate this term from abstract and discussion.

++++++++++++++++++++++++++++++++++++++++++++++++++++++++++++++++++++++++++++++++++++

[17] - Driving forces are diminished?

"This can reduce synaptic currents in distal dendrites, as greater local depolarization during EPSPs limits synaptic driving forces at the peak of the EPSP”.

” ... conditions in which synaptic driving forces are diminished (see Figures 2 and 8)”.

We do not see any graphical pointers or represents of diminished driving forces in Figure 2.

The authors should provide clean, simple and sufficient explanations on each point they want to make, with figure enhancements (arrows) and auxiliary sentences.

+++++++++++++++++++++++++++++++++++++++++++++++++++++++++++++++++++++++++++++++++++++

[18] - Proximal dendrites improve synaptic currents at any given location??

"On the other hand, the increased variability of EPSP-spike coupling when barrages were initiated at different RMPs in proximal dendrites reflects the non-linear amplification of synaptic responses with increased depolarization, leading to a greater range of synaptic currents across voltage at any given location”.

Non-linear amplification of synaptic responses with increased depolarization is not something that happens in proximal dendrites only. I do not understand the significance of “proximal dendrites” in this sentence. “Proximal dendrites improve synaptic currents at any given location”??

What is the meaning of this sentence, and how this sentence improves our understating of the process by which “NMDA receptors stabilize synaptic integration”?

+++++++++++++++++++++++++++++++++++++++++++++++++++++++++++++++++++++++++++++++++++++

[19] - NMDA conductances oppose the impact of reduced synaptic driving force

"Similarly, by opposing the impact of reduced synaptic driving force on AMPA-mediated currents, voltage-dependent NMDA conductances can reduce the variability of total synaptic current during temporal integration of clustered excitatory inputs”.

Many believe that NMDA conductances do not oppose the problem with reduced driving force, instead they make it worse.

Could you please explain in the manuscript how: “NMDA conductances oppose the impact of reduced synaptic driving force”?

*

********

CRITIQUE ENDS HERE

********

## Author Response

Response to Reviewer Comments for eN-NWR-0396-20

We have read and reread the reviewer comments below several times, and tried to interpret them in the most charitable way possible. Below we offer a point-by-point response. Where appropriate, we have modified the manuscript as noted, with changes designated by blue text. For the many comments that we perceive as frivolous, nonconstructive, and/or nonprofessional, we provide detailed rebuttals below.

Comment 1.1: The reviewer seems to be willfully misconstruing our use of the word “synergism”. We use that word only to explain how the voltage-dependance AND slow kinetics of NMDA conductances act synergistically to lower the variability of synaptic spike thresholds (or thresholds for any arbitrary subthreshold voltages) for discrete spatiotemporal patterns of input delivered across multiple locations or voltages. This is a completely independent concept relative to the interaction of AMPA and NMDA conductances at individual synapses, or at multiple synapses within a given dendritic area (both of which have been well described before). We are extraordinarily clear in the text that our results focus solely on the novel and not-necessarily-intuitive finding that the reduction in somatic response variability to spatiotemporal patterns of input generated across all possible dendritic locations and initial membrane potentials depends on interaction of both properties of NMDA receptors (voltage-dependence and slow kinetics), and does not occur when either property is expressed alone. This is “synergy.”

Comment 1.2.a: The reviewer is using a spurious semantic argument to conflate the term “spatial domains”. Branco et al. (2010) did not test “across spatial domains", they looked instead at individual dendritic spans (similar to our 50 um spans) and tested the impact of patterned activation of spines existing in that single domain (e.g., sequential activation of inputs in one direction or the other). They did not attempt to compare identical spatiotemporal patterns of synaptic input delivered to different spatial domains across a dendritic tree, and also did not measure this across a large number of initial membrane potentials. Further, they did not quantify the variability of somatic responses to different, but statistically similar, spatiotemporal patterns of input before and after blockade of NMDA receptors. Thus, their work, while important (and cited in our original and revised manuscripts), does not amount to “overlap” of scientific discovery.

The novelty of our study is that we rigorously test the impact of synaptic conductance on the variability of somatic responses to identical spatiotemporal patterns of input delivered across all possible dendritic locations and across a large variety of initial membrane potentials. This has not previously been done. To our knowledge, the nearest finding to ours is in Larkum et al. (2009), where (as mentioned in our original manuscript) simulations “found the threshold number of synapses necessary for initiating an apical trunk calcium spike to be relatively consistent across tuft locations when NMDA conductances were present, but that thresholds for AMPA-only inputs grew exponentially, and to non-physiological synaptic densities, with distance from the initiation zone in the apical dendrite.”

Comment 1.2.b: Again, the reviewer conflates the term “spatial domain”. Further, EPSP “amplification” is not the point of our paper. Regardless of any amplification (or dampening), we are measuring the somatic fidelity (see below) of input-output functions for spatiotemporal patterns of synaptic input across dendritic domains (50 um spans at 10 um intervals in our tests) and RMP. We recognize the contributions of Connelly et al. (2016) by citing them in the original draft and revised draft of the manuscript, just as we continue to cite the review article by Major et al. (2013). Our novel result, that somatic responses to spatiotemporal patterns of input delivered to different dendritic locations and/or across different initial membrane potentials, lead to less variable somatic responses and EPSP-spike coupling when NMDA and AMPA conductances are co-localized at synapses, does not overlap with the findings of either paper.

Comment 1.2.c: Again, the reviewer seems to be purposefully conflating aspects of synaptic transmission. We make no original claims about NMDA receptors generating larger events at distal locations. This is indeed well known for decades, and we cite many of the fundamental papers that revealed this location-dependent impact of NMDA conductance. As we make abundantly clear to our readers, the novel finding of the present paper is that the co-expression of AMPA and NMDA conductances at synapses greatly reduces the variability of EPSP-spike coupling for spatiotemporal pattens of input delivered to different dendritic domains and/or at different initial membrane potentials.

Comment 1.2.d: We make no novel claim for that finding a relationship between NMDA conductance and local input impedance. This effect is well known, and we cite relevant papers to that effect. The reviewer appears to be working hard to find “overlap” where none exists.

Comment 1.2.e: Again, we make no claims about “discovering” that NMDA currents are larger in high-impedance compartments. We recognize that they do, and cite relevant papers showing as much, even as we note that this particular feature of NMDA conductances is what underlies the very different (and previously unreported) finding that combined synaptic AMPA and NMDA conductances reduce the variability of somatic responses to spatiotemporal patterns of synaptic input delivered at different dendritic locations.

Comment 1.2.f: There is no overlap. Magee and Cook (2000), the paper we believe the reviewer is pointing to, showed that AMPA conductances increase with distance along the apical dendrite of CA1 neurons (and only for CA1 neurons; see for instance, Williams and Stuart, 2002, for an alternative result in layer 5 cortical neurons), and therefore somatic responses to individual synaptic activations tended to be of similar size at the somatic due to increased AMPA receptor conductance at distal synapses having more AMPA receptors. This result is completely unrelated to the findings in our study. To be clear, we show that, for spatiotemporal patterns of input delivered to different dendritic locations and/or across different initial membrane potentials, summed EPSPs and/or EPSP-spike coupling are less variable when uniform NMDA and AMPA conductances are co-localized at synapses. Even as the Magee and Cook result is completely unrelated to any of our findings or simulations, we continue to cite their important work as an example of the many mechanisms by which neurons can reduce somatic variability in synaptic integration.

Comment 1.3a: There is no overlap. We make no claims about “integration windows” or how timing influences NMDA conductances. Perhaps the reviewer is concerned about our results shown in Figure 1, where we show for single EPSPs that NMDA conductances are larger at distal locations. If so, the reviewer is mistaken if he thinks we present Figure 1 as original findings. Figure 1 exists simply to show that our model works as expected given the many decades of study of the interaction of dendritic location (i.e., input impedance) and synaptic conductances. As in our original draft (emphasis ours), we start the section with the sentence, “As expected, given the cable properties of dendrites (Gulledge et al., 2005), the amplitude and kinetics of the resulting EPSPs depended on synaptic conductance type, RMP, and the dendritic location of synaptic input (Figure 1A).”

Comment 1.3.b: Again, there is no overlap. The reviewer seems to willfully conflate the term “AMPA/NMDA ratio”. In the review by Major et al. (2013), they discuss AMPA/NMDA ratios in relation to thresholds necessary to evoke NMDA spikes at individual synapses. We make no claim about AMPA/NMDA ratios in regard to NMDA spike thresholds, but include Figure 7 simply to show that the novel result we find throughout the paper (namely that co-localization of AMPA and NMDA conductances reduce the variability of somatic voltage responses and EPSP-spike coupling in response to spatiotemporal patterns of synaptic input generated at different dendritic locations and/or different initial membrane potentials) is not an artifact of using one particular AMPA:NMDA ratio. This is simply a control experiment to show that the “effect” on somatic response variability is robust across a variety of physiologically relevant AMPA:NMDA ratios.

Comment 1.3.c: Again, there is no overlap. We do not test “time jitter” on synaptic integration of AMPA or NMDA components of inputs. Similarly, Major et al. (2013) do not examine the impact of combined AMPA and NMDA conductances on the variability of somatic responses to spatiotemporal patterns of synaptic input generated at different dendritic locations and/or different initial membrane potentials. Repeatedly throughout the manuscript we clearly communicate the novel and previously unreported result that combining AMPA and NMDA conductances reduces the variability of somatic responses to spatiotemporal patterns of synaptic input generated at different dendritic locations and/or different initial membrane potentials. It is not our responsibility to point out all of the other research papers that have not tested for, and therefore not reported, this finding. While AMPA:NMDA ratios have been measured many times in a variety of systems to study various aspects of synaptic transmission and integration (especially with regard to synaptic plasticity), in no system have AMPA:NMDA ratios been tested in regard to their impact on the variability of somatic responses to spatiotemporal patterns of synaptic input generated at different dendritic locations and/or different initial membrane potentials.

Comment 1.4: As noted above, we included Figure 1 simply to show that our model acts “rationally” and as expected given the well-established knowledge regarding cable properties of dendrites. We do not suggest that these results are “new” or unexpected. To the contrary, we state clearly in the text that they are “as expected”.

Comment 1.5: In our manuscript, we do not claim to have “discovered” that NMDA spikes are larger or longer in duration when occurring in smaller dendrites. This has been known for over two decades, much in advance of the Gao et al. (2020) paper in Journal of Neurophysiology (see for instance the many papers by Jackie Schiller and Matthew Larkum). Regardless, the question is not whether NMDA-mediated responses are larger at distal locations. Our paper tests whether location-dependent increases in NMDA current, combined with the reduced AMPA currents occurring in distal dendrites (due to driving force reductions during the EPSP; see below) reduces the variability of somatic responses to spatiotemporal patterns of input delivered at locations across the dendritic tree and/or across initial membrane potential. This finding is new and previously unreported.

Comment 2: The reviewer again appears to be willfully conflating issues. The action potential waveform has no impact on the results of our study, as we are only measuring the thresholds number of synapses necessary to generate an action potential. Consider the following aspects of our study:

a) We use the occurrence of an action potential only to identify synaptic spike thresholds.

b) Action potentials are not necessary to show this increased fidelity of synaptic integration, as in Figure 6 we used arbitrary somatic “thresholds” far below action potential threshold.

c) Voltage-gated conductances, in the axon or anywhere else, are not necessary for demonstrating the reduced variability of synaptic integration, as Figure 8 is conducted in a purely passive neuron.

Finally, we would point out that we not attempting to simulate “pyramidal neurons”. Our goal is to explore the “synergistic” effect of AMPA and NMDA receptors on somatic response fidelity across a wide range of neuron morphologies.

Comment 3: We disagree. Figure 5 is important as it shows the effect sizes of results in Figure 4. As is evident from Table 2 (which lists effect sizes for results in Figure 4E only), a list of such effect sizes across all dendritic sizes, combinations of conductances for location-specific vs voltage specific responses would be unwieldy. Figure 5 allows one to graphically see how effect sizes for the various conductances are influenced by dendritic location and initial membrane potential.

Comment 4: The middle panel is important in showing that there is a dose dependence to replacing some of the AMPA conductance with a voltage-dependent and/or slow conductance. Thus, there is a real “effect” and not simply large uncorrelated variability from which we happen to choose conductance values that show an “effect”. Further, the results from the neuron with a 200-μm dendrite on a larger neuron is important to show as it demonstrates that the apparent effect of dendrite length on the robustness of NMDA-AMPA interactions is not real, but instead reflects differences in overall electrotonic size of the neurons. By including a larger neuron with a 200-μm dendrite (from Figure 3A) we show that the reduced variability of somatic responses to spatiotemporal patterns of synaptic input generated at different dendritic locations and/or across different initial membrane potentials when AMPA and NMDA receptors are co-expressed occurs even in small (200 μm) dendrites when those dendrites are attached to larger neurons. Not including these data would give the false impression that the presence of NMDA receptors will not enhance the fidelity of EPSP-spike coupling in small dendrites.

Comment 5: While we appreciate the reviewers attempt to help improve our writing, our original text concisely and clearly conveys our results. We see no improvement, and indeed a significant reduction of clarity, in the reviewer’s contribution. We will leave it to the editor to decide whether our writing style is up to the standards of eNeuro.

Comment 6: We are unclear what the reviewer is suggesting. We clearly define the unit of effect size (sAMPA-only) on page 7 (lines 153-164). There is no need to use ambiguous “units” in the text.

Comment 7: As one gets very close to threshold (but remains “just-subthreshold” even at the most excitable dendritic location) there can be significant lift by sodium conductances in the AIS and soma that generates the small peaks of concern to the reviewer. We now explain the origin of these peaks in the Figure 2 legend.

Comment 8]: The reviewer is wrong. The patterns for the different conductances are the same, but each conductance has a different number of “just-subthreshold” synaptic activations within that pattern (based on their different synaptic thresholds for action potential generation). We now provide clear explanation of this on page 9 (lines 218-221) and in the Figure 2 legend (see below).

Comment 9: The reviewer is incorrect. There are no missing data, and all traces for all conductances at all locations (at 20 μm intervals) are shown in Figure 2B.

Comment 10: We apologize for the confusion. We now clarify in the text and figure legend that Figure 2B shows responses to identical subthreshold patterns of synaptic input across all dendritic locations so that the gradual increase in NMDA conductance across location can be visualized. We have added the following text to the Results section on page 9, lines 218-221: “This can best be seen in Figure 2B, which shows somatic and local dendritic responses to identical patterns of subthreshold input (determined for each conductance type as the maximal number of synapses that remained subthreshold across all dendritic locations) in a neuron with an RMP of -70 mV.” We also add the following text to the Figure 2 legend: “While the spatiotemporal pattern is identical for all synaptic conductances, the number of synapses activated is adjusted for each conductance such that the maximal number of synaptic inputs that remain subthreshold across all dendritic locations is delivered. When occurring at locations where they are “just-subthreshold", somatic EPSPs can recruit somatic and axonal voltage-gated sodium conductances, producing the small peaks visible on the largest somatic EPSPs.”

Comment 11: We now clarify on page 8 (line 202) that we tested the impact of combining AMPA and NMDA conductances on the fidelity of EPSP-spike coupling in models with dendrites of various lengths.

Comment 12: The reviewer is wrong. When NMDA receptors are present, synaptic currents (Isyn) increase naturally with distance because the NMDA conductance is voltage dependent. This can clearly be seen in the currents in Figure 2B, but is also extremely well known as the underlying reason why NMDA spikes have lower synaptic thresholds at distal dendritic compartments even when the number of AMPA and NMDA receptors are held constant at synapses across dendritic space. This does not happen with AMPA receptors that lack voltage dependence (also shown in Figure 2B), where synaptic currents actually become smaller due to the collapse in driving force during the EPSP (where Eq-Vm becomes smaller). We feel we have explained these basic physiological principles adequately in the manuscript.

Comment 13: We thank the reviewer for noticing this error. We have now corrected the text to refer to Figure 6C.

Comment 14: We have added a reference to Figure 7A on page 17 (line 455).

Comment 15: The convergence is clear and inescapable. As AMPA conductances are decreased, the CV increases toward the level of NMDA-only inputs (which is depicted as a static horizontal line, as it is independent of AMPA conductance values). Common sense dictates that, as AMPA conductances approach zero, CVs for AMPA+NMDA conductances will converge with the NMDA only conductance (as NMDA conductance is all that remains). It is also clearly evident in Figure 7C as the green symbols curve up toward the NMDA only values as AMPA conductance decreases.

Comment 16: Merriam-Webster defines “Fidelity” as “the degree to which an electronic device (such as a record player, radio, or television) accurately reproduces its effect (such as sound or picture)”. This is analogous to what we are measuring by our CV of synaptic thresholds throughout the paper (i.e., the “fidelity” of EPSP-spike coupling).

Comment 17: We are not testing for, and do not graph, changes in driving force. We expect that the vast majority of readers of eNeuro understand that driving forces are diminished when the membrane potential approaches the synaptic reversal potential, as occurs during EPSPs preferentially in high-impedance dendritic compartments.

Comment 18: We thank the reviewer for this comment. We no longer include the term “at proximal dendrites", and provide on page 19 (lines 523-530) a more elaborate explanation for why the “fast-NMDA” conductance increases synaptic threshold CVs primarily in proximal dendrites.

Comment 19: We believe the reviewer is referencing to the sentence, “Similarly, by opposing the impact of reduced synaptic driving force on AMPA-mediated currents, voltage-dependent NMDA conductances can reduce the variability of total synaptic current during temporal integration of clustered excitatory inputs” (lines 562-565). The sentence is true as written. Reductions in driving force during large-amplitude EPSPs (typical of high-impedance dendritic compartments) act to reduce AMPA currents (synaptic current = driving force * synaptic conductance; see Figure 2B, and notice how the AMPA currents become smaller with distance from the soma). However, because the NMDA conductance is voltage dependent, synapses that also include NMDA receptors have a dynamic conductance that increases with depolarization (i.e., when Vm is closer to Esyn, and driving forces are diminished). This acts to maintain a relatively substantial current even as driving force collapses (see Figure 2B, where AMPA+NMDA currents increase with distance from the soma).
